# Airway epithelial cGAS inhibits LPS-induced acute lung injury through CREB signaling

**DOI:** 10.1038/s41419-023-06364-0

**Published:** 2023-12-19

**Authors:** Zhangchu Jin, Zhehua Shao, Shiyi Yang, Anyi Guo, Yinling Han, Yinfang Wu, Yun Zhao, Yanping Wu, Jiaxin Shen, Min Zhang, Xueqin Zhan, Wenqi Diao, Songmin Ying, Chao Zhang, Wen Li, Huahao Shen, Zhihua Chen, Fugui Yan

**Affiliations:** 1 Key Laboratory of Respiratory Disease of Zhejiang Province, Department of Respiratory and Critical Care Medicine, Second Affiliated Hospital of Zhejiang University School of Medicine, Hangzhou, 310009 Zhejiang China; 2 Department of Pulmonology, Children’s Hospital, Zhejiang University School of Medicine, National Clinical Research Center for Child Health, National Children’s Regional Medical Center, Hangzhou, 310009 Zhejiang China; 3 International Institutes of Medicine, the Fourth Affiliated Hospital of Zhejiang University School of Medicine, Yiwu, 322000 China; 4 Department of Pharmacology and Department of Respiratory and Critical Care Medicine of the Second Affiliated Hospital, Zhejiang University School of Medicine, Key Laboratory of Respiratory Disease of Zhejiang Province, Hangzhou, 310009 China; 5 Department of Anatomy, Zhejiang University School of Medicine, Hangzhou, 310058 China; 6 State Key Lab of Respiratory Disease, National Clinical Research Center for Respiratory Disease, Guangzhou, 510120 China

**Keywords:** Acute inflammation, Infectious diseases

## Abstract

Increased levels of cytosolic DNA in lung tissues play an important role in acute lung injury. However, the detailed mechanisms involved remain elusive. Here, we found that cyclic GMP-AMP synthase (cGAS, a cytosolic DNA sensor) expression was increased in airway epithelium in response to increased cytosolic DNA. Conditional deletion of airway epithelial cGAS exacerbated acute lung injury in mice, cGAS knockdown augmented LPS-induced production of interleukin (IL)-6 and IL-8. Mechanically, deletion of cGAS augmented expression of phosphorylated CREB (cAMP response element-binding protein), and cGAS directly interacted with CREB via its C-terminal domain. Furthermore, CREB knockdown rescued the LPS-induced excessive inflammatory response caused by cGAS deletion. Our study demonstrates that airway epithelial cGAS plays a protective role in acute lung injury and confirms a non-canonical cGAS-CREB pathway that regulates the inflammatory responses in airway epithelium to mediate LPS-induced acute lung injury.

## Introduction

Acute respiratory distress syndrome (ARDS) is a syndrome of acute respiratory failure caused by non-cardiogenic pulmonary edema. The mortality of ARDS is approximately 25–40% in most studies [1, 2]. Despite many years of research, there is still no effective pharmacotherapy for this syndrome. Therefore, it is urgent to study the pathogenesis of ARDS in order to identify novel targeted therapies.

Airway epithelial cells are considered as the primary responders to respiratory insults [3], and are involved in the regulation of lung inflammation. Various microorganisms or cell-injury associated endogenous molecules bind to pattern recognition receptors such as Toll-like receptors on the lung epithelium and activate the innate immune system [4]. This process generates reactive oxygen species, leukocyte proteases, chemokines, and cytokines that help eliminate pathogens but can also exacerbate lung injury [5]. Several studies have demonstrated that airway-epithelial-cell-derived hepcidin [6], nuclear factor erythroid 2-related factor 2 (Nrf2) [7], and lipoxin A4 [8] play an important role in the pathogenesis of acute lung injury. However, critical functions of airway epithelial cells for coordinating the innate immune response in the development of acute lung injury have not been fully identified.

Cyclic guanosine monophosphate (GMP)-adenosine monophosphate (AMP) synthase (cGAS) is a newly discovered sensor that detects cytosolic DNA as a universal danger-associated molecular pattern (DAMP) [9]. As an enzyme, cGAS is activated upon binding to exogenous or endogenous cytosolic DNA. It then produces cyclic-GMP-AMP (cGAMP) and activates a downstream signaling molecule called stimulator of interferon genes (STING), which then recruits tank binding kinase 1 (TBK1) and phosphorylates interferon regulatory factor 3 (IRF3) to induce the transcription of type I interferons (IFNs) and other cytokines [10]. As a key pattern recognition receptor, cGAS plays a key role in the initiation and regulation of innate immunity and is expected to become a potential therapeutic target for cancer [11], systemic lupus erythematosus [12], and infectious diseases [13].

We have recently shown that cGAS deletion in airway epithelial cells significantly attenuates an ovalbumin-induced or house dust mite-induced T helper 2 (TH2) immune response in mice [14]. However, whether airway epithelial cGAS also mediates the pathogenesis of acute lung injury remains unclear. By contrast, in the pathogenesis of ARDS caused by severe infection, invading microorganisms cause damage to the mitochondrial or nuclear genome of lung tissue cells and promote the release of free DNA fragments into the cytosol, so as to trigger an inflammatory reaction and eventually lead to the formation of acute lung injury [15]. However, so far, the detailed mechanism of acute lung injury induced by increased cytosolic DNA from airway epithelium remains elusive.

In the current study, we hypothesized that airway epithelial cGAS initiated inflammatory reactions and mediated acute lung injury by sensing cytosolic DNA induced by endotoxin. Indeed, we found that lipopolysaccharide (LPS) triggered cytosolic DNA release and augmented cGAS expression in airway epithelial cells. However, surprisingly, conditional deletion of airway epithelial cGAS significantly exacerbated rather than attenuated LPS-induced acute lung injury. Furthermore, we found a non-canonical cGAS-CREB pathway that specifically regulated the inflammatory responses of airway epithelium to mediate acute lung injury. Our findings suggest that an airway epithelial cGAS-CREB pathway could play a protective role in LPS-induced acute lung injury.

## Materials and methods

### Reagents

RNAiso plus (9109), reverse transcription reagents (DRR037A), and SYBR Green Master Mix (DRR041A) were from Takara Biotechnology (Japan). LPS (L2880) and doxycycline (D9891) was purchased from Sigma-Aldrich. G150 (HY-128583) and 666-15 (HY-101120) was purchased from MedChemExpress (USA). Antibodies to cGAS (#15102, #31659), CREB (#9197), p-CREB (#9198) and γH2AX(#80312) were purchased from Cell Signaling Technology (Danvers, MA). Antibodies to cGAS (26416-1-AP) was purchased from Proteintech (USA). Antibodies to dsDNA (ab27156), CC10 (ab40873) and RPA (ab2175) was purchased from Abcam (USA). Antibodies to ACTB(AC026) was purchased from ABclonal (USA). Mito-Tracker (C1049), antibodies to Anti-DDDDK-Tag (AF5051) and Anti-HA(AF2305) were purchased from Beyotime (China). Antibodies to cGAS (sc-515777) and small interfering RNAs (siRNA) of control (sc-37007), cGAS (sc-95512) and STING (sc-92042) were purchased from Santa Cruz Biotechnology. SiRNA of CREB (SR306377) was purchased from Origene (USA). ELISA kits were purchased from LIANKE BIO (China). All primers were synthesized by Sangon Biotech, Shanghai. PCR primers used in our experiments are shown in Table 1.

**Table 1:** Primer sequences used for experiments.

Genes	Primer (5′-3′)	Assay
β-Actin (h)	Forward: 5′-CATGTACGTTGCTATCCAGGC-3′	RT-PCR
	Reverse: 5′-CTCCTTAATGTCACGCACGAT-3′	
Il6(h)	Forward: 5′-ACTCACCTCTTCAGAACGAATTG-3′	RT-PCR
	Reverse: 5′-CCATCTTTGGAAGGTTCAGGTTG-3′	
Il8(h)	Forward: 5′-CACCATGACTTCCAAGCTGGC-3′	RT-PCR
	Reverse: 5′-TTATGAATTCTCAGCCCTCTTC-3′	
cGAS(h)	Forward: 5′-GGGAGCCCTGCTGTAACACTTCTTAT-3′	RT-PCR
	Reverse: 5′-CCTTTGCATGCTTGGGTACAAGGT-3′	
Creb(h)	Forward: 5′-CCCTGCCCAACCCTACAATG-3′	RT-PCR
	Reverse: 5′-CCCTGCCCAACCCTACAATG-3′	
β-Actin (m)	Forward: 5′-AGAGGGAAATCGTGCGTGAC-3′	RT-PCR
	Reverse: 5′-CAATAGTGATGACCTGGCCGT-3′	
Il6(m)	Forward: 5′-CTGCAAGAGACTTCCATCCAG-3′	RT-PCR
	Reverse: 5′-AGTGGTATAGACAGGTCTGTTGG-3′	
Cxcl1(m)	Forward: 5′-ACCCAAACCGAAGTCATA-3′	RT-PCR
	Reverse: 5′-GGTGCCATCAGAGCAGT-3′	
Cxcl2(m)	Forward: 5′-CCCAGACAGAAGTCATAGC-3′	RT-PCR
	Reverse: 5′-TCCTTTCCAGGTCAGTTA-3′	
Tnf-α(m)	Forward: 5′-CTGAACTTCGGGGTGATCGG-3′	RT-PCR
	Reverse: 5′-GGCTTGTCACTCGAATTTTGAGA-3′	
Il-1β(m)	Forward: 5′-TTCAGGCAGGCAGTATCACTC-3′	RT-PCR
	Reverse: 5′-GAAGGTCCACGGGAAAGACAC-3′	
cGAS^flox/flox^(m)	Forward: 5′-CCAGAATTAGGAAATTAACCCC-3′	Genotype identification
	Reverse: 5′-GCCAGGTGACACAACATCC-3′	
CC10(m)	Forward: 5′-ACTGCCCATTGCCCAAACAC-3′	Genotype identification
	Reverse: 5′-AAAATCTTGCCAGCTTTCCCC-3′	
tetO(m)	Forward: 5′-TGCCACGACCAAGTGACAGCAATG-3′	Genotype identification
	Reverse: 5′-AGAGACGGAAATCCATCGCTCG-3′	

*h* human, *m* mouse

### Mice

*cGAS*^*flox/flox*^ mice (C57BL/6 background) were purchased from Nanjing Biomedical Research Institute of Nanjing University (Jiangsu, China). *CC10-rtTA/(tetO)7-Cre* transgenic mice (C57BL/6 background) transgenic mice (C57BL/6 background) were provided by Dr. Y. Ke (Zhejiang University School of Medicine).

*CC10-rtTA/(tetO)7-Cre/cGAS*^*flox/flox*^ mice were generated by crossing the *cGAS*^*flox/flox*^ mice with *CC10-rtTA/(tetO)7-Cre* transgenic mice. Age- and sex-matched littermate animals (*CC10-rtTA/cGAS*^*flox/flox*^, *(tetO)7-Cre/cGAS*^*flox/flox*^, and *cGAS*^*flox/flox*^) were used as controls of above mice in the experiments. To induce the activation of Cre recombinase in transgenic mice, 6–8 weeks old mice were fed with doxycycline in their drinking water (2 mg/ml) for 20 days before establishing the model of LPS-induced acute lung injury. After confirming the knockdown effect, the *CC10-rtTA/(tetO)7-Cre/cGAS*^*flox/flox*^ mice treated with doxycycline were then designated as AE-cGAS^△/△^ mice.

The genomic DNA from tails or lungs was extracted for genotyping. C57BL/6 mice (18–20 g) were purchased from the Animal Center of Slaccas (Shanghai, China). All animal experiments were in accordance with the Guide for the care and use of laboratory animals (https://www.nap.edu/catalog/12910/guide-for-the-care-and-use-of-laboratory-animals-eighth), and were approved by the Animal Care and Use Committee at Zhejiang University (License number: ZJU20210305).

### Animal models

To establish a mouse model of LPS-induced acute lung injury, cages of mice were randomly allocated to groups and were exposed to LPS (in 50 μl saline) at a dose of 5 mg/kg by intratracheal instillation for 24 h. Meanwhile, control mice were treated with the same volume of saline.

For pulmonary edema assessment, the lung tissues were dried in a 60 °C oven for 72 h until constant weight. The lung wet to dry (W/D) ratio was recorded to assess the condition of pulmonary edema. Lung vascular permeability was assessed by Evans blue accumulation in the lung tissue. Evans blue dye (20 mg/kg, i/v) was injected 2 h before termination of the experiment. The quantitative analysis of Evans blue labeled albumin extravasation was performed by spectrophotometric analysis of Evans blue extracted from the lung tissue samples [16]. Survival rate was tracked every 24 h after LPS instillation.

### Cell culture and LPS treatment

HBE cells were purchased from American Type Culture Collection (CRL-2741), and were cultured in RPMI 1640 (Gibco, C11875500BT) with 10% FBS (Gibco, 10082147) and 1% penicillin-streptomycin (Beyotime Biotechnology) in 5% CO^2^ at 37 °C. After LPS (100 ug/ml) stimulation for 24 h, cells were then subjected to further analysis. U2SO and HEK293T cells were cultured in DMEM (Sigma, D5796) with 10% FBS and 1% penicillin-streptomycin in 5% CO^2^ at 37 °C. All cell lines were regularly tested for mycoplasma infection.

### Plasmid design

The coding sequences (CDS) of cGAS and CREB were obtained from Miaoling Corporation, China. The full-length gene sequence of wild-type human cGAS was synthesized and subcloned into a pcDNA3.1 vector, along with a N-terminal Flag tag (DYKDDDDK) as a cGAS fusion protein to facilitate protein expression. An HA tag (YPYDVPDYA) was inserted on the C-terminal domain of CREB. All cGAS truncate mutants were generated by a PCR-based method and were confirmed by DNA sequencing.

### RNA knockdown and plasmid transfection

siRNA knockdown of cGAS, STING and CREB in HBE cells (GenMute™ siRNA Transfection Reagent, SignaGen Laboratories, siRNA concentration: 10 nM) were handled following the manufacturer’s protocols. As a negative control, a non-targeting scrambled siRNA pool (control siRNA) was used at the same concentration. The recombinant plasmids of cGAS and CREB, which were truncated sequences, were transfected into HEK293T and U2SO cells (PolyJet™ In Vitro DNA Transfection Reagent, SignaGen Laboratories) following the manufacturer’s protocol. Knockdown and overexpression efficiency were assessed by measuring RNA levels by quantitative real-time PCR or protein levels by immunoblotting.

### Immunoprecipitation

The cells were collected and suspended in 500 μl lysis buffer (Beyotime, P0013) containing a protease inhibitor cocktail (1 μl/100 μl, Biomake), and then incubated under gentle agitation on ice for 30 min, followed by centrifugation at 12,000 rpm for 30 min at 4 °C. Supernatant (40 μl) was mixed with lysis buffer plus the loading buffer as the input sample. The remaining supernatants were immunoprecipitated with anti-Flag beads, anti-HA beads or magnetic protein A/G beads (Beyotime, P2108) conjugated with anti-cGAS (Santa, sc-515777) or anti-CREB (CST, #9197) antibodies. After overnight incubation, beads were washed four times with PBST buffer (PBS with 0.1% Tween-20), then boiled with 70 μl loading buffer at 100 °C for 10 min. Proteins were applied to the indicated SDS-PAGE, and analyzed by western blot.

### Immunoblotting

Protein was extracted from cells or lung tissues using RIPA buffer (Beyotime, P0013C) with protease and phosphatase inhibitors (Roche Diagnostics GmbH, 04906845001). Samples were electrophoresed through 6–15% polyacrylamide gels and immunoblotted with the relevant antibodies using standard methods. Signal was detected by chemiluminescence using ChemiDoc Touch Imaging System.

### Quantitative real-time PCR

HBE cells and lung homogenates were lysed with RNAiso plus (Takara Biotechnology, 9109), and total RNA was extracted. By using reverse transcription reagents (Takara Biotechnology, DRR037A), RNAs were reverse-transcribed. Then, the expression of genes was measured by quantitative real-time PCR, which was performed on a StepOne real-time PCR system (Applied Biosystems, Foster City, CA) using SYBR Green Master Mix (Takara Biotechnology, DRR041A). All protocols were performed following the manufacturer’s instructions.

### ELISA

Cell culture medium samples were centrifuged at 1000 rpm for 10 min, and the supernatants were collected and stored at −80 °C. The concentration of IL-6 and IL-8 in cell culture supernatants and mouse cytokines such as CXCL1, CXCL2, IL-6, TNF-α, or IL-1β in homogenate of lung tissue were measured by ELISA kits (MultiSciences, LiankeBio) following the manufacturer’s instructions.

### cGAMP concentration determination

HBE cells were washed with PBS twice and then treated directly with lysis buffer (M-PER™, Thermo Scientific). Cells were scraped to dislodge them from the plate surface and cells should be inspected to ensure lysis. Centrifuge the samples at ≥600 g at 4 °C for 15 min and assay the supernatant directly by 2’,3’-Cyclic GAMP Enzyme Immunoassay Kit (Arbor Assays, K067-H1/H5) following the manufacturer’s instructions.

### BALF collection and analysis

Mice were sacrificed 24 h after the last exposure to LPS, and the lungs were lavaged by injection of 0.4 ml PBS into the lungs and the collection of cells (completed three times). The total number of BALF cells was counted, then the remaining BALF was centrifuged (400 g for 10 min at 4 °C). The supernatant was retained for further analysis, while the cell pellet was resuspended in PBS and centrifuged on glass slides. Then the cells on the glass slides were stained with Wright–Giemsa stain (Baso, BA-4017), and differential counts were assessed by counting 200 total cells.

### Histological analysis

After exposure to LPS, the lungs were removed and fixed in 4% paraformaldehyde at 4 °C for 24 h. After fixation, the lungs were embedded in paraffin for hematoxylin & eosin (H&E) analysis. Stained slices were visualized using an Olympus BX51 microscope equipped with a 4/0.3 NA objective and a DP70 digital camera. The inflammatory score was calculated as the following features: lung interstitial edema, hemorrhage, and neutrophil infiltration. Each feature could be assessed with a score of 0 (no injury), 1 (limited injury), 2 (visible injury), and 3 (severe injury). The scores of these three aspects were added to produce a total score from 0 to 9 [17]. Three random fields of each lung sections were assessed and scores were averaged to record for the analysis.

### Immunofluorescence staining

Paraformaldehyde-fixed and paraffin-embedded lung sections were prepared and immunostained for CC10 antibody (Abcam, ab40873), cGAS antibody (Santa, sc-515802) and p-CREB antibody (CST, #9198) following standard methods. Images were photographed using an Olympus BX53 inverted microscope (Olympus, Melville, NY). HBE cells were fixed and stained with dsDNA antibody (Abcam, ab27156), cGAS antibody (Proteintech, 26416-1-AP), P-CREB antibody (CST, #9198), γH2AX antibody (CST, #80312) and RPA (Abcam, ab2175) at 4 °C for 8 h. The abundance of cytosolic dsDNA was quantified double blind by the number of positive dots per cell in high-power fields (10–20 cells per experiment). The relative fluorescence intensity of P-CREB was measured with ImageJ software, and the mean relative fluorescence intensities were normalized to the levels of controls. The γH2AX and RPA positive cells were manually counted in three random fields and calculated the percentage.

### Proximity Ligation Assay (PLA)

The PLA was performed using the Duolink In Situ Red Starter Kit Mouse/ Rabbit (Sigma, DUO92101) according to the manufacturer’s instructions. Briefly, HBE cells were seeded in a 12-well chamber slide. The following day, cells were washed twice with PBS, fixed with 4% formaldehyde for 15 min and permeabilized with 0.1% Triton X-100 in PBS for 10 min. The samples were blocked, and then incubated overnight with antibodies against cGAS and CREB. The following day, the samples were incubated with the MINUS and PLUS PLA probes corresponding to the primary antibodies used, followed by ligation with circle-forming DNA oligonucleotides and rolling-circle amplification to generate the PLA signal. Finally, the samples were mounted with DAPI-containing Fluoromount-G™ (SouthernBiotech). The slides were imaged with a confocal microscope (Olympus BX53).

### RNAseq

The samples were disposed and analyzed by BGI (Shenzhen). RNAseq data had been uploaded in NCBI under BioProject. ID: GSE185523.

### Molecular docking procedure

Molecular docking was performed to investigate the binding mode between cGAS and CREB using the ZDOCK server (zdock.umassmed.edu). The three-dimensional (3D) structure of full-length CREB and cGAS were built by AlphaFold. For docking, the default parameters were used as described in the ZDOCK server. The top ranked pose as judged by the docking score was subject to visual analysis using PyMoL 1.7.6.

### Statistics

Sample sizes were based on preliminary data and previous experience. The investigator was blinded to the group allocation for the assessment of histology but was not blinded for other experiments. No samples were excluded from analysis. Data were tested for normality and presented as means ± SEM. Statistical analysis was performed with GraphPad Prism (GraphPad software, San Diego, California, USA). Differences between two groups were identified using the Student *t* test and multiple groups using one- or two-way ANOVA with Sidaks multiple comparisons. *P* values of less than 0.05 were considered statistically significant.

## Results

### LPS triggered cytosolic DNA release and induced cGAS expression in airway epithelium

**Fig. 1:**
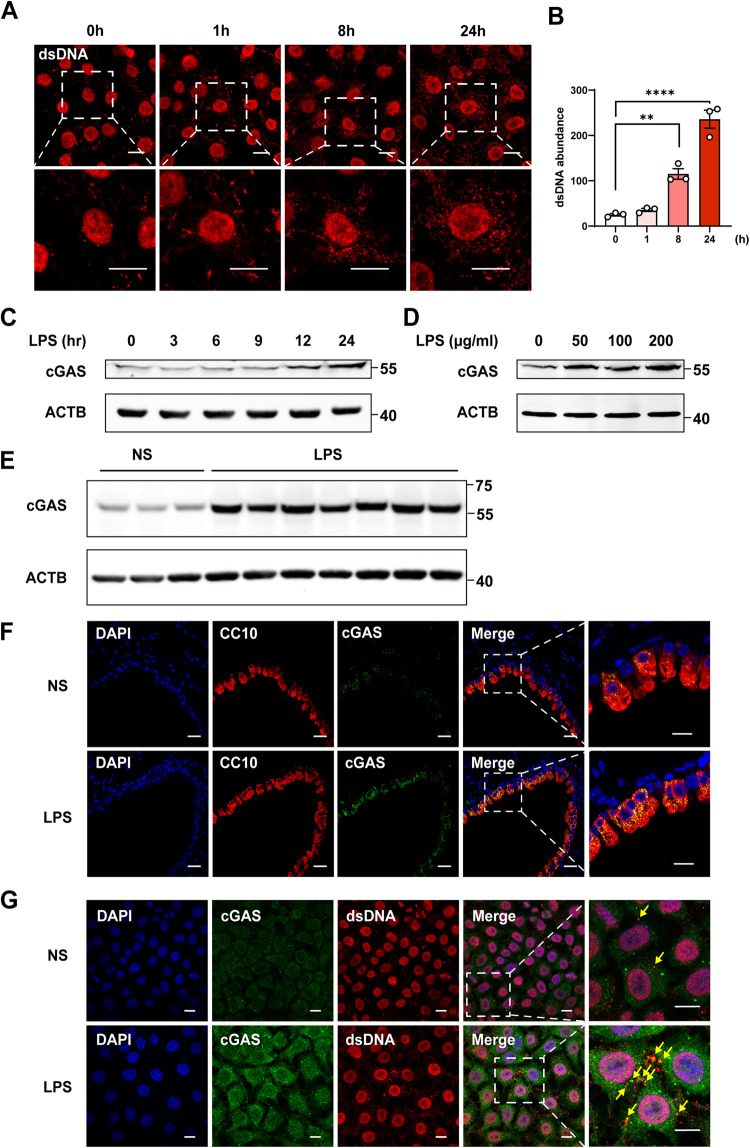
cGAS is upregulated in the airway epithelium following LPS treatment in vivo and in vitro. Representative immunofluorescence images of dsDNA (red) (**A**) and quantification of dsDNA abundance (**B**) in HBE cells stimulated with LPS (100 µg/ml) for 0, 1, 8, and 24 h. *n* = 3. Scale bars, 10 μm. **C** Immunoblot assays of cGAS in HBE cells treated with LPS (100 μg/ml) for 0, 3, 6, 9, 12, and 24 h. **D** Immunoblot assays of cGAS in HBE cells were treated with LPS (50, 100, 200 μg/ml) for 24 h. **E** Immunoblot assays of cGAS in lung tissues of C57BL/6 mice after LPS (5 mg/kg) or saline intratracheal instillation for 24 h. **F** Representative immunofluorescence images of cGAS (green), nuclei (DAPI, blue), and airway epithelial cells (with CC10, red) in lung tissues of C57BL/6 mice after LPS (5 mg/kg) or saline intratracheal instillation for 24 h. Scale bars, 20 μm. **G** Representative immunofluorescence images of the co-localization of cGAS (green) and dsDNA(red) in HBE cells stimulated with LPS (100 µg/ml) for 24 h. Scale bars, 10 μm. In **B**, the data presented are one representative experiment of three independent experiments and shown as mean ± SEM. Statistical analyses were calculated using One-way ANOVA with Sidaks multiple comparisons. **p < 0.01, ****p < 0.0001.

A previous study had shown that LPS could induce DNA fragments to be released into the cytosol of endothelial cells and activate cGAS [18], so we explored the effects of LPS on levels of cytosolic DNA and cGAS in airway epithelial cells. As shown in Fig. 1A, B, LPS markedly increased the cytosol double-stranded DNA (dsDNA) contents of human bronchial epithelial (HBE) cells in a time-dependent manner. To further determine the primary source of cytosolic dsDNA, we conducted an examination of HBE cells that were stained with dsDNA and mitotracker (mitochondria). Our findings indicated that the cytosolic dsDNA primarily originated from the mitochondria (Supplementary Fig. S1A). Additionally, we assessed the expression levels of γH2AX and RPA, which are recognized as classic markers of nuclear DNA damage [19–21]. Our observations revealed that there was no significant increase in either γH2AX or RPA in the HBE cells treated with LPS (Supplementary Fig. S1B–F). Taken together, we concluded that the mitochondria, rather than the nucleus, served as the principal origin of cytosolic dsDNA. Additionally, LPS significantly induced cGAS protein expression of HBE cells in a time-dependent and dose-dependent manner (Fig. 1C, D). Furthermore, cGAS levels were also markedly increased in the lung tissues of a mouse model of LPS-induced acute lung injury (Fig. 1E), and immunofluorescence staining showed that cGAS was highly expressed and mainly located in the airway epithelial cells of the mice (Fig. 1F). To provide evidence of dsDNA sensed by cGAS, we used confocal microscopy images of HBE cells stained with anti-dsDNA and anti-cGAS antibodies. As shown in Fig. 1G, cGAS was co-located with dsDNA in the cytoplasm, and the percentage of co-localization was significantly increased in LPS-induced HBE cells compared with control cells.

### Knockdown of cGAS augmented LPS-induced inflammatory response in HBE cells

**Fig. 2:**
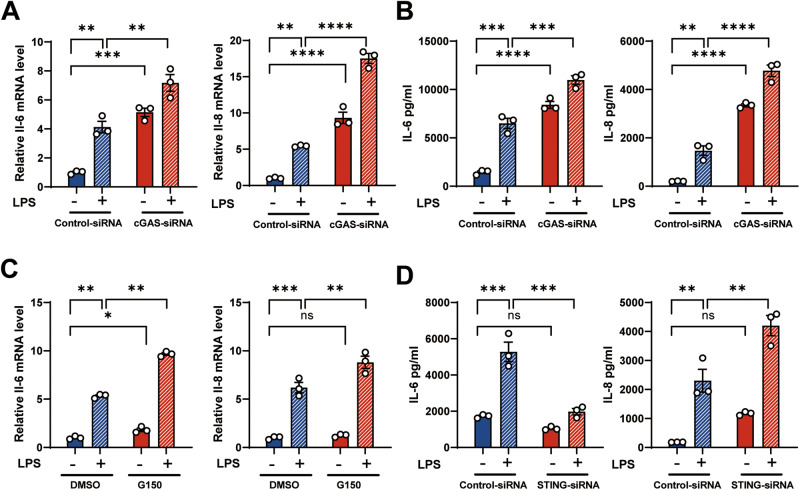
cGAS deficiency augments the inflammatory response in airway epithelium. **A**, **B**, **D** HBE cells were transfected with control-siRNA, cGAS-siRNA or STING-siRNA for 24 h and incubated with 100 μg/ml LPS for an additional 24 h. **A** RT-qPCR of IL-6(left) and IL-8 (right) in HBE cells (*n* = 3). **B** IL-6 and IL-8 ELISA analysis of culture supernatants from HBE cells (*n* = 3). **C** RT-qPCR of IL-6 (left) and IL-8 (right) in HBE cells treated with G150 (cGAS inhibitor, 20 μM) or DMSO and stimulated with LPS (100 µg/ml) for 24 h (*n* = 3). **D** IL-6 and IL-8 ELISA analysis of culture supernatants from HBE cells (*n* = 3). In **A**–**D**, the data presented are one representative experiment of three independent experiments and shown as mean ± SEM. Statistical analyses were calculated using two-way ANOVA with Sidaks multiple comparisons. ns no significance, *p < 0.05, **p < 0.01, ***p < 0.001, ****p < 0.0001.

Previous studies have confirmed that cGAS promotes the overproduction of inflammatory factors [22, 23]. We next focusd on identifying the biological function of airway epithelium-derived cGAS upon LPS exposure. cGAS was known to catalyze the synthesis of cGAMP, which subsequently activated downstream signaling [9]. Our observations demonstrated a significant decrease in cGAMP levels in cGAS-knockout cells (Supplementary Fig. S2A). However, unexpectedly, we showed that knockdown of cGAS markedly augmented interleukin (IL-6) and IL-8 production in basic condition and LPS-induced HBE cells (Fig. 2A, B, Supplementary Fig. S2B). G150, as a specific inhibitor of cGAS, also augmented the IL-6 and IL-8 mRNA expression in LPS-induced HBE cells (Fig. 2C). As STING is a downstream molecule of cGAS, we also explored the role of STING in LPS-induced inflammation. Unexpectedly, knockdown of STING promoted IL-8, but significantly inhibited IL-6 production in LPS-induced HBE cells (Fig. 2D, Supplementary Fig. S2B). These results suggest that cGAS has a protective role in the LPS-induced inflammatory response and this process is not fully dependent of STING.

### Conditional deletion of airway epithelial cGAS significantly exacerbated LPS-induced acute lung injury in mice

**Fig. 3:**
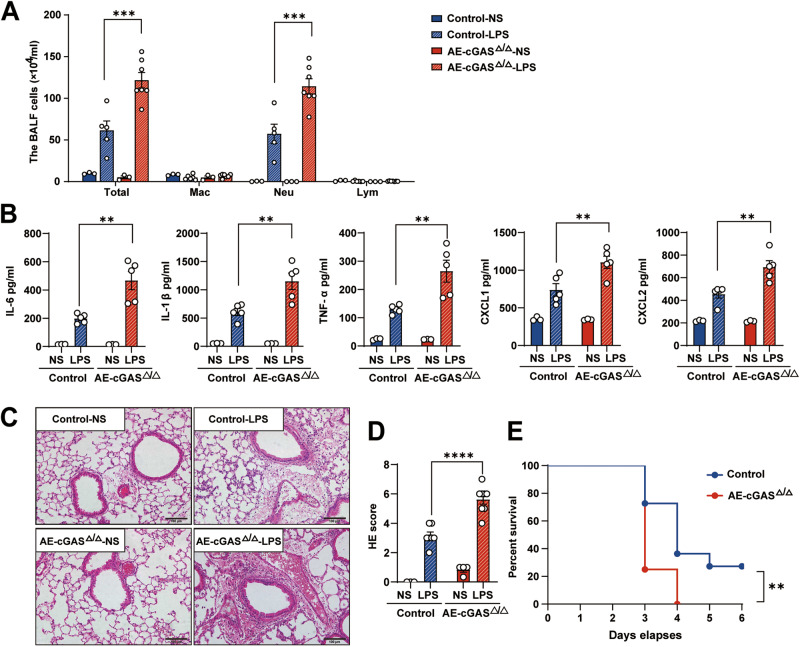
Conditional deletion of cGAS in airway epithelium exacerbates LPS-induced lung injury in vivo. **A**–**D** *CC10-rtTA/(tetO)7-Cre/cGAS*^*flox/flox*^ mice and their littermate controls were fed with doxycycline in their drinking water (2 mg/ml) for 20 days (designated as AE-cGAS^△/△^ mice) and then intratracheally challenged with LPS (5 mg/kg) for 24 h. **A** The count of the inflammatory cells in BALF from mice. **B** The protein levels of inflammatory cytokines IL-6, IL-1β, TNF-α, CXCL1, and CXCL2 in lung tissues analyzed by ELISA. **C** Representative lung tissue with H&E staining from mice. **D** Histological damage score from H&E images. **E** Survival of AE-cGAS^△/△^ mice (*n* = 12) and their littermate controls (*n* = 11) after LPS (20 mg/kg) intratracheal instillation. In **A**, **B** and **D**, each symbol represents an individual mouse (*n* = 3~7); Two-way ANOVA with Sidaks multiple comparisons (**A**, **B**, **D**); log-rank survival analysis (**E**). The data presented are one representative experiment of three independent experiments and shown as mean ± SEM. **p < 0.01, ***p < 0.001, ****p < 0.0001.

We confirmed that cGAS knockdown augmented LPS-induced inflammatory factors in HBE cells. Next, the role of cGAS in a mouse model of LPS-induced acute lung injury was explored. Deletion of cGAS in the airway epithelial cells was achieved by crossing floxed cGAS mice (*cGAS*^*fl/fl*^) with *CC10*-rtTA/(tetO)_7_-Cre mice (Supplementary Fig. S3). In the LPS-induced acute lung injury mouse model, conditional deletion of airway epithelial cGAS significantly increased the numbers of bronchoalveolar lavage fluid (BALF) total cells and neutrophils (Fig. 3A). Additionally, levels of inflammatory factors in lung tissues, including IL-6, IL-1β, TNF-α, CXCL-1, and CXCL-2 (mRNA as well as proteins), were markedly increased in LPS-induced lung homogenates from AE-cGAS^△/△^ mice compared with those in littermate control mice (Fig. 3B, Supplementary Fig. S4A). Consistently, aggravated inflammatory cell infiltration was observed in lung tissues of AE-cGAS^△/△^ mice by hematoxylin and eosin staining, and the average hematoxylin and eosin score was increased from 3.1 in littermate control mice to 5.6 in AE-cGAS^△/△^ mice (*P* < 0.05, Fig. 3C, D). These results suggest that airway epithelial cGAS prevents the excessive activation of LPS-induced pulmonary inflammation in mice.

Having determined the effects of cGAS deletion in LPS-induced pulmonary inflammation, we then explored whether cGAS deletion had the same efficacy for LPS-induced vascular permeability, pulmonary edema, and mortality. As shown in Supplementary Fig. S4B, conditional deletion of airway epithelial cGAS increased the Evans blue contents in BALF in AE-cGAS^△/△^ mice, which suggested an exacerbation of vascular permeability. Pulmonary edema, as evidenced by the lung wet/dry weight ratio, was also increased at 24 h post-LPS exposure in AE-cGAS^△/△^ mice (Supplementary Fig. S4C). Furthermore, airway epithelial cGAS deletion exacerbated LPS-induced mortality in mice, as the survival rate of AE-cGAS^△/△^ mice was significantly decreased after LPS exposure compared with that of littermate control mice (*P* = 0.0075, Fig. 3E).

### cGAS knockdown augmented phosphorylated CREB expression

**Fig. 4:**
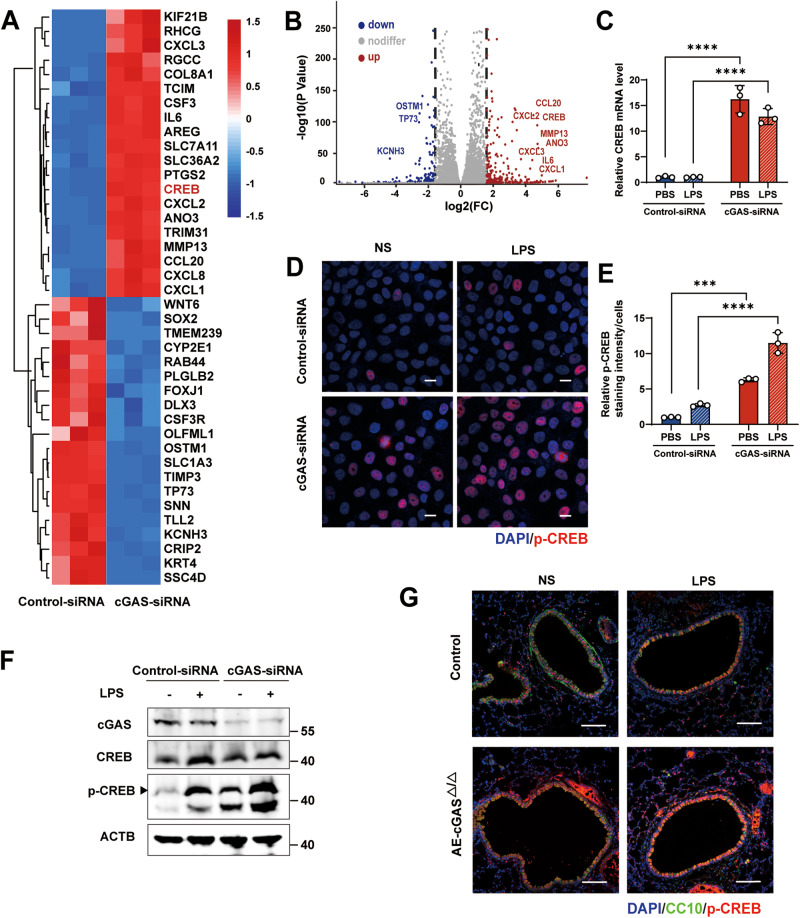
cGAS deficiency increases CREB expression and phosphorylation in HBE cells. **A** The heat map of the differentially expressed genes (DEGs) (top 20 up-regulated and downregulate genes) in control- or cGAS-siRNA HBE cells through RNA sequencing analysis. Blue indicates a relatively low expression and red indicates a relatively high expression. **B** The volcano plot of differentially expressed genes (DEGs) in control- or cGAS-siRNA HBE cells through RNA sequencing analysis. **C** RT-qPCR of CREB in HBE cells transfected with cGAS siRNA or control siRNA for 24 h and incubated with 100 μg/ml LPS for an additional 24 h (*n* = 3). Representative immunofluorescence images (**D**) and quantification of staining (**E**) of P-CREB (red) and nuclei (DAPI, blue) in HBE cells transfected with cGAS siRNA or control siRNA for 24 h and incubated with 100 μg/ml LPS for an additional 24 h. Scale bars, 10 μm. **F** HBE cells were transfected with cGAS siRNA or control siRNA for 24 h and were treated with LPS (100 μg/ml) for 24 h, and the cell lysates were collected for immunoblotting. **G** Representative lung immunofluorescence images of P-CREB (red), nuclei (DAPI, blue), and airway epithelial cells (marked with CC10, green) in mice. Scale bars, 100 μm. Two-way ANOVA with Sidaks multiple comparisons (**C**, **E**), ***p < 0.001, ****p < 0.0001. The data presented are one representative experiment of three independent experiments and shown as mean ± SEM.

To further explore the detailed molecular mechanisms of cGAS inhibiting the LPS-induced inflammatory response in HBE cells, we further analyze transcriptional differences in RNA sequencing analysis and the data showed that the expression of 7686 genes in HBE cells were altered after cGAS knockdown (3850 upregulated and 3836 downregulated), and the top 50 upregulated genes mainly included cancer-related genes, some chemokines, and transcription factors. Among them, CREB mRNA levels were found markedly upregulated in cGAS small interfering RNA (siRNA)-transfected HBE cells (Fig. 4A, B). Previous studies have shown that CREB can combine with transcription sites of IL-6 and IL-8 genes and promote their expression [24, 25]. Therefore, we performed further quantitative PCR to confirm the results of CREB expression (Fig. 4C). Both western blot analysis and immunofluorescence staining showed that knockdown of cGAS markedly increased the expression of phosphorylated CREB in LPS-induced HBE cells (Fig. 4D–F). Consistent with our in vitro studies, conditional deletion of cGAS in airway epithelium also significantly increased phosphorylated CREB expression in the airway of mice (Fig. 4G).

### cGAS directly interacted with CREB

**Fig. 5:**
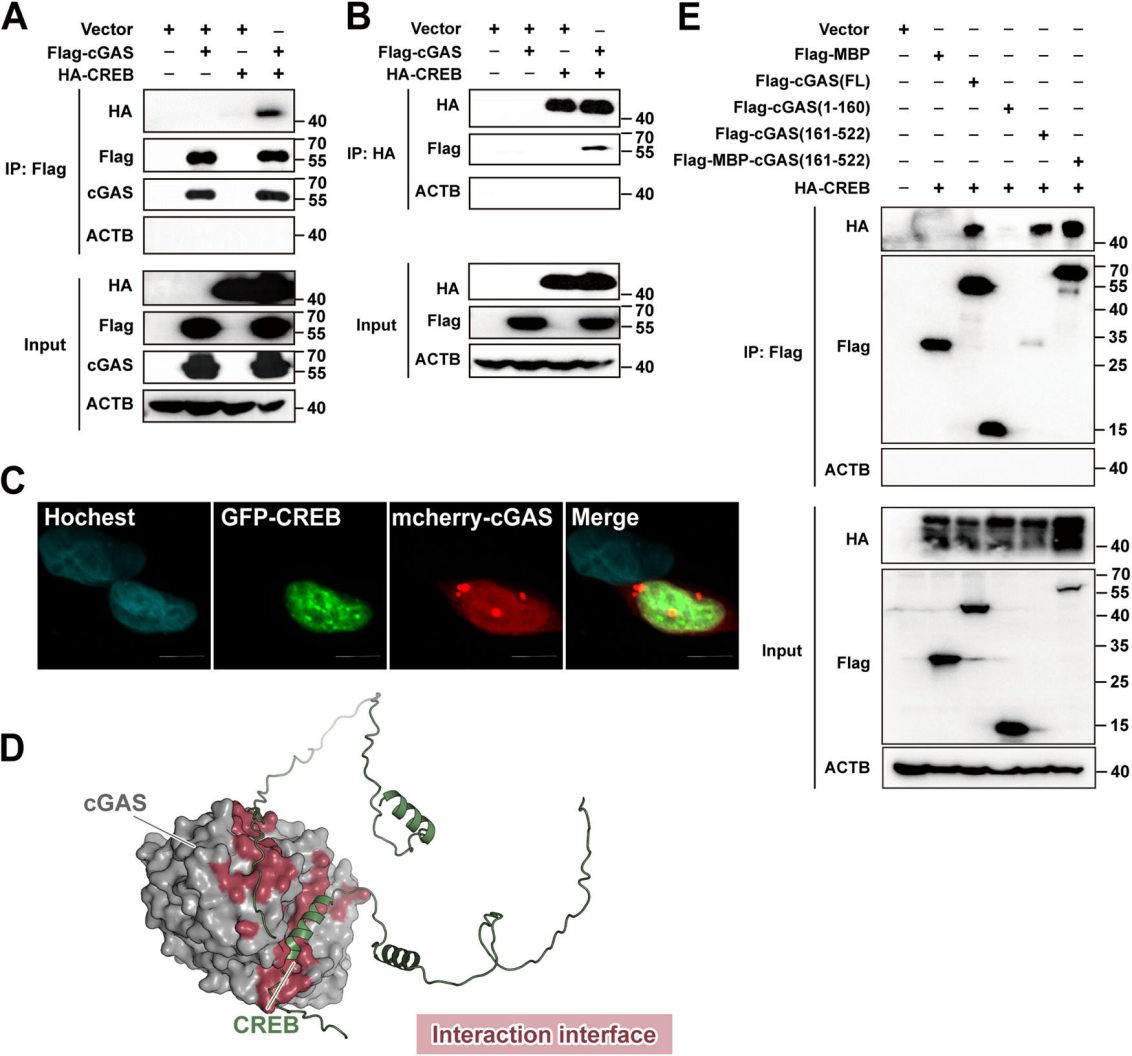
cGAS binds directly to CREB. **A**, **B** HEK293T cells were transfected with Flag-labeled cGAS, together with HA-labeled CREB for 48 h. Cell lysates were immunoprecipitated with anti-Flag (**A**) or anti-HA (**B**) and subjected to immunoblotting. **C** Representative immunofluorescence images of U2SO cells transfected with mCherry-labeled cGAS, together with GFP-labeled CREB for 48 h. Scale bars, 10 μm. **D** Docking model of CREB and the C-terminal domain of the cGAS complex. CREB was shown as ribbon diagram in green. The C-terminal domain of cGAS was shown as a gray surface. The interaction areas were highlighted in pink. The structure of cGAS and CREB were predicted by AlphaFold. **E** HEK293T cells were transfected with Flag-labeled MBP, Flag-labeled cGAS (FL), Flag-labeled cGAS (1–160), Flag-labeled cGAS (161–522), Flag-MBP labeled cGAS (161–522) together with HA-labeled CREB for 48 h. Cell lysates were immunoprecipitated with anti-Flag and subjected to immunoblotting.

To investigate the underlying molecular mechanisms by which cGAS inhibits CREB hyperphosphorylation, we tested the interaction between cGAS and CREB by co-immunoprecipitation. Plasmids encoding hemagglutinin (HA)-tagged CREB and Flag-tagged cGAS were co-transfected into HEK 293 T cells. Cell lysates were precipitated with anti-Flag antibody sequentially, followed by western blotting with anti-HA antibody. Anti-HA immunoprecipitated Flag-cGAS and anti-Flag immunoprecipitated HA-CREB (Fig. 5A, B). Immunofluorescence for cGAS and CREB showed their co-localization (Fig. 5C). Consistent with the overexpression data, endogenous CREB coprecipitated with cGAS, demonstrating a direct physical interaction between these two proteins (Supplementary Fig. S5A, B). cGAS was reported to be constitutively present in the nucleus and cytosol [26–28]. To further validate the co-localization of cGAS with CREB, proximity ligation assays (PLA) were performed. The presence of red fluorescence in the nucleus indicated the co-localization of cGAS and CREB within the cellular nucleus (Supplementary Fig. S5C).

Furthermore, molecular docking analysis predicted an interface between CREB and the C-terminal domain of cGAS (Fig. 5D). Since the C-terminal domain of cGAS alone did not express well in cells, we constructed the cGAS C-terminus fused to the maltose binding protein (MBP) for further study. Co-immunoprecipitation analyses showed that HA-CREB could be co-immunoprecipitated by Flag-cGAS, Flag-cGAS (161–522), and Flag-cGAS (161–522)-MBP, but not Flag-cGAS (1–160). Furthermore, Flag-cGAS (161–522)-MBP but not MBP alone precipitated HA-CREB (Fig. 5E). These data provide strong evidence that cGAS binds to CREB via its C-terminal domain.

### CREB knockdown rescued the LPS-induced excessive inflammatory response caused by cGAS deletion

**Fig. 6:**
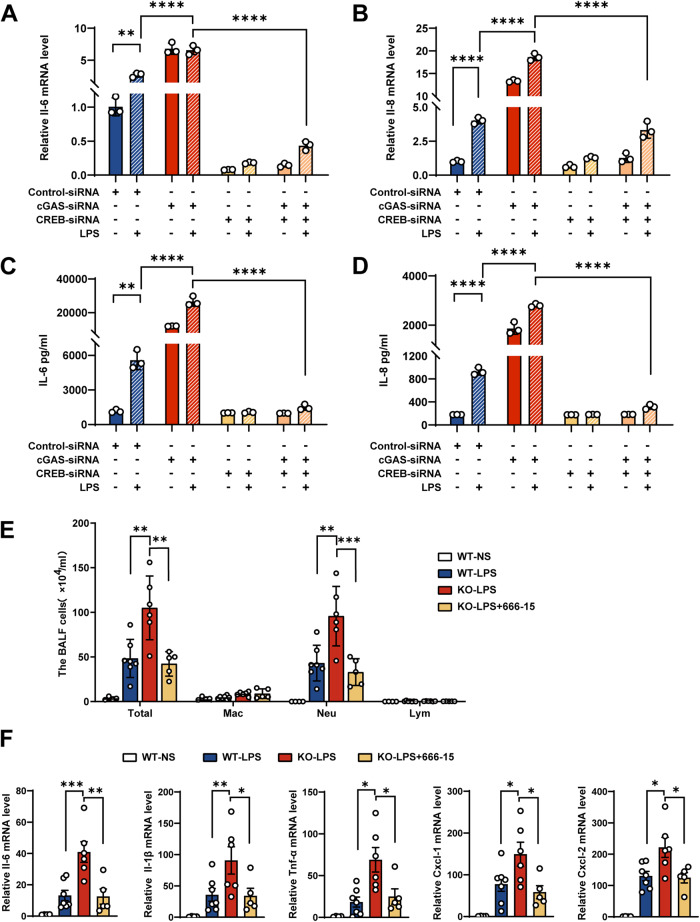
Targeting CREB in vitro reduces the inflammatory response in HBE cells. **A**–**D** HBE cells transfected with cGAS-, CREB- or control-siRNA were incubated with 100 μg/ml LPS for 24 h. RT-qPCR (**A**, **B**) and ELISA (**C**, **D**) analysis of IL-6 and IL-8 of HBE cells and culture supernatants were performed. The data presented are one representative experiment of three independent experiments and shown as mean ± SEM and statistical analyses were calculated using two-way ANOVA with Sidaks multiple comparisons. **E**, **F** *CC10-rtTA/(tetO)7-Cre/cGAS*^*flox/flox*^ mice (KO) and their littermate controls (WT) were fed with doxycycline in their drinking water (2 mg/ml) for 20 days. The mice were pretreated with 10 mg/kg 666-15(CREB inhibitor) or DMSO for 2 h and then intratracheally challenged with LPS (5 mg/kg) for 24 h. **E** The count of the inflammatory cells in BALF from mice. **F** RT-qPCR of IL-6, IL-1β, TNF-α, CXCL1, and CXCL2 in lung tissues from mice. In **E** and **F**, each symbol represents an individual mouse (*n* = 4~7). The data shown as mean ± SEM and statistical analyses were calculated using one-way ANOVA. *p < 0.05, **p < 0.01, ***p < 0.001, ****p < 0.0001.

Since cGAS regulates the LPS-induced inflammatory response through the CREB signaling pathway, both CREB siRNA and cGAS siRNA were transfected into LPS-induced HBE cells. As shown in Fig. 6A–D, CREB siRNA completely rescued the IL-6 and IL-8 over-production caused by cGAS knockdown in LPS-induced HBE cells. 666-15 is a potent and selective CREB inhibitor (IC50 = 81 nM) [29, 30]. The addition of 666-155 significantly alleviated the impact of cGAS-knockdown in LPS-stimulated HBE cells (Supplementary Fig. S6A–C). Additionally, we applied 666-15 in cGAS epithelial knockout (cGAS cKO) animal models. It was observed that cGAS cKO models exhibited an increased neutrophil count and expression of inflammation factors. Nevertheless, the levels of neutrophil count and inflammation factors expression were reduced in cGAS cKO models upon the addition of 666-15 (Fig. 6E, F). Taken together, our findings suggest that under physiological conditions, cGAS inhibits the LPS-induced inflammatory response by suppressing the hyperphosphorylation of CREB.

## Discussion

In this study, we demonstrated that LPS triggered cytosolic DNA release and increased cGAS expression in airway epithelium, and conditional deletion of airway epithelial cGAS significantly exacerbated LPS-induced acute lung injury in mice. Furthermore, we elucidated the detailed molecular mechanism of airway epithelial cGAS regulating acute lung injury, which is a non-canonical cGAS-CREB pathway that specifically regulates the inflammatory responses of airway epithelium to mediate LPS-induced acute lung injury.

In contrast to the traditional cGAS-STING signal pathway, we used mass spectrometry and co-immunoprecipitation to confirm a non-canonical cGAS-CREB pathway that plays an important role in the regulation of inflammation. Our results indicated that cGAS knockdown markedly increased the expression of CREB. Previous studies [31] have confirmed that CREB can enhance the gene transcription of cGAS. Our results suggested that cGAS knockdown can lead to a feedback increase in CREB expression to compensate for the decrease of cGAS levels. It was widely acknowledged that p-CREB represented the active form of CREB [32, 33]. Subsequent western blot analysis revealed an elevated level of p-CREB in cGAS knockdown cells (Fig. 4F). Although no specific sites of cGAS affecting CREB phosphorylation have been identified yet, our results have shown that the C-terminal domain of cGAS interacts directly with CREB. This interaction might generate steric hindrance for protein phosphatase binding, resulting in a decreased level of CREB phosphorylation. Besides, the conventional view recognized cGAS as a cytoplasmic protein. Recent reports challenged this simplistic view by showing that cGAS could be found within the nucleus [27]. Our study also indicated that the cGAS localized in the nucleus could interacted directly with CREB.

Several recently published studies have focused on the crucial role of cGAS in acute lung injury [34–38]. Compared with findings in mice with global cGAS deficiency [34], mice with airway epithelial cGAS deficiency were used in our study and revealed a completely opposing conclusion. Namely, conditional deletion of airway epithelial cGAS augmented LPS-induced acute lung injury, rather than the protective effect caused by global cGAS deletion. Additionally, several mouse studies have shown that cGAS knockdown will cause a decrease in the host’s resistance to tuberculous bacteria [39], Legionella [40], Pseudomonas aeruginosa [41], and herpes simplex virus (HSV) [42], resulting in a significant increase in the load of corresponding pathogens in mice. This finding is similar to the results of our study. However, it must be admitted that after cGAS knockout of airway epithelium in this study, the increased degree of pulmonary edema in mice with acute lung injury was not as significant as inflammation. This may be related to the characteristic of the mouse model of acute lung injury induced by LPS, that is, the pulmonary edema of this model is only mild [43]. Moreover, cGAS-knockdown cells showed an increased level of inflammatory cytokines expression as compared with corresponding wild-type cells. And this phenomenon not observed in mouse models. This may be related to differences between the in vitro and in vivo conditions. Importantly, the effects of cGAS-knockdown upon LPS stimulation was consistent in vivo and vitro, indicating that cGAS played a protective role in acute lung injury. Our RNA sequencing results showed that the top 50 significantly upregulated genes in LPS-stimulated HBE cells with cGAS knockdown included TBXT, SPP1, EPB41L3, GALC, and RPS4Y1. Most of these genes are related to the onset and invasion of various tumors [44, 45], but have no obvious effect on the expression of inflammatory factors. Additionally, previous studies have confirmed that cGAS induces autophagy via STING trafficking and drives noncanonical-inflammasome activation [46, 47]. Since autophagy and the inflammasome both play an important role in the expression of inflammatory factors, we also wanted to know whether they were involved in the upregulation of LPS-induced inflammatory factors caused by cGAS knockdown. However, our RNA sequencing data revealed that the expression level of autophagy and inflammasome-related genes did not change significantly after cGAS knockdown.

cGAS usually works through downstream STING, Allen et al. showed that IL-6 production caused by extracellular vesicles in macrophages was dependent on cGAS-STING [48]. However, our in vitro research showed that knockdown of STING significantly inhibited upregulation of IL-6 induced by LPS in HBE cells, which was opposite to the effect of knockdown of cGAS. Therefore, our study suggests that cGAS does not dependent on STING to inhibit LPS-induced inflammation. Indeed, cGAS not only mediates the synthesis of type I interferon through the classical STING signal pathway but also plays a key role independently of STING in the process of liver injury [49], colon cancer development [50], and the suppression of genomic instability [51]. Our study further expands the functions of cGAS and suggests that cGAS acts as a brake necessary to regulate LPS-induced inflammatory response of airway epithelium. It has the potential to become a new therapeutic target for acute lung injury caused by pathogen infections.

### Supplementary information

Supplementary Materials and Figures

Original western blots

checklist

## Data Availability

All datasets generated and analyzed during this study are included in this published article and its Supplementary Information files. Full length uncropped western blots are available in the supplemental material files. Additional data are available from the corresponding author on reasonable request.
