# Supplementary material for: Airway epithelial cGAS inhibits LPS-induced acute lung injury through CREB signaling
Source: Cell Death Dis. 2023 Dec 19;14(12):844. doi: 10.1038/s41419-023-06364-0 (PMC10730695; doi:10.1038/s41419-023-06364-0)
Supplement: Supplementary file 1 — Supplementary Materials and Figures [file 41419_2023_6364_MOESM1_ESM.pdf]

Supplementary Materials and Figures

Figure S1

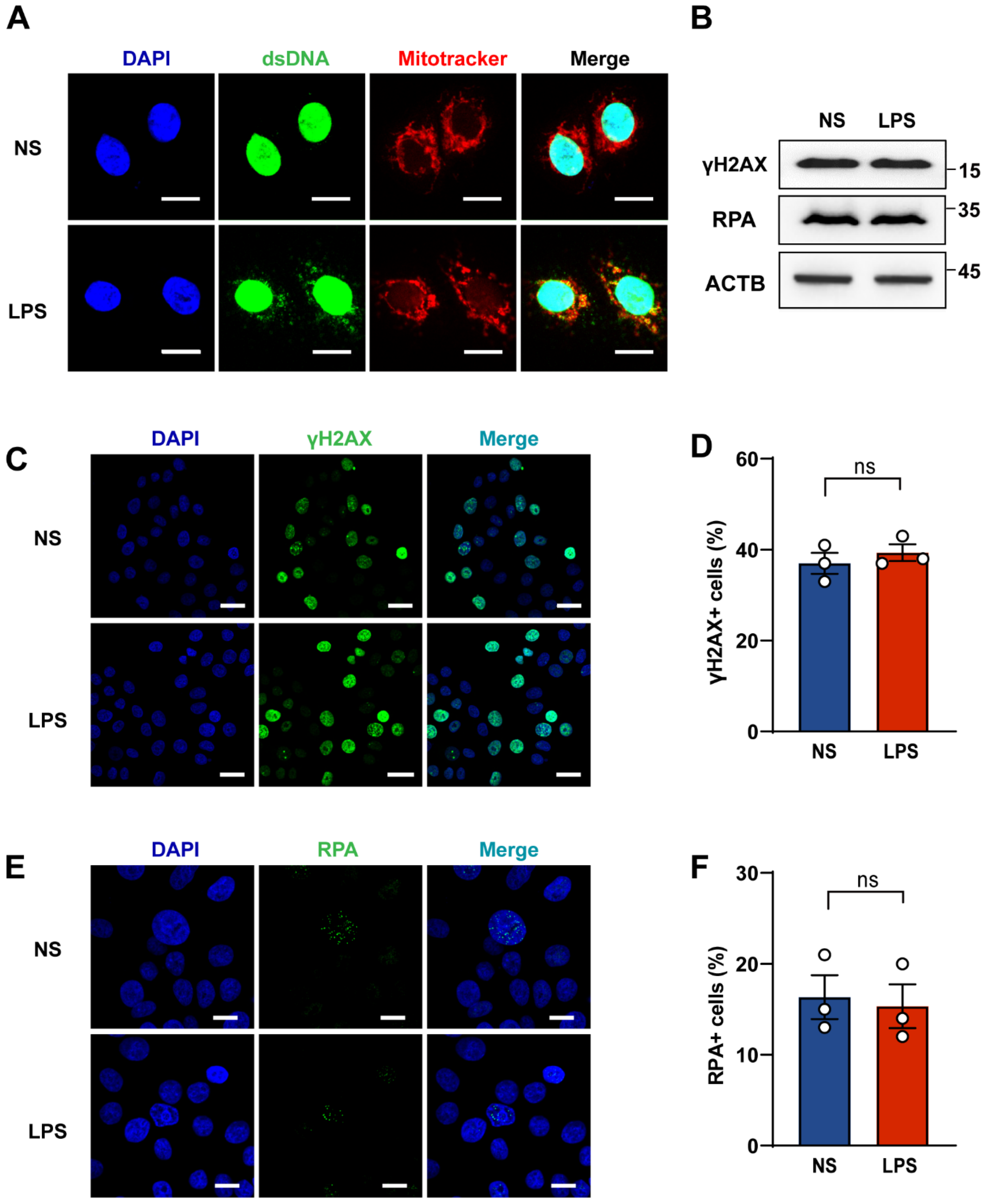

Figure S1. Cytosolic dsDNA induced by LPS originates from mitochondria.

A) Representative immunofluorescence images of dsDNA (green) and Mitochondria (red) in HBE cells stimulated with LPS (100 µg/ml) for 24 h. Scale bars, 10 µm. **B**) Immunoblot assays of γH2AX and RPA in HBE cells treated with LPS (100 µg/ml) for 24 h. **C**) Representative immunofluorescence images of γH2AX

(green) in HBE cells stimulated with LPS (100 μg/ml) for 24 h. Scale bars, 20 μm. **D**) Percentage of γH2AX+ cells (%) of cells described in (C) was calculated (n=3). **E**) Representative immunofluorescence images of RPA (green) in HBE cells stimulated with LPS (100 μg/ml) for 24 h. Scale bars, 10 μm. **F**) Percentage of RPA+ cells (%) of cells described in (E) was calculated (n=3). In (D), (F), the data presented are one representative experiment of three independent experiments and shown as mean ± SEM. Statistical analyses were calculated using a two-tailed unpaired Student's t test. ns, no significance.

Figure S2

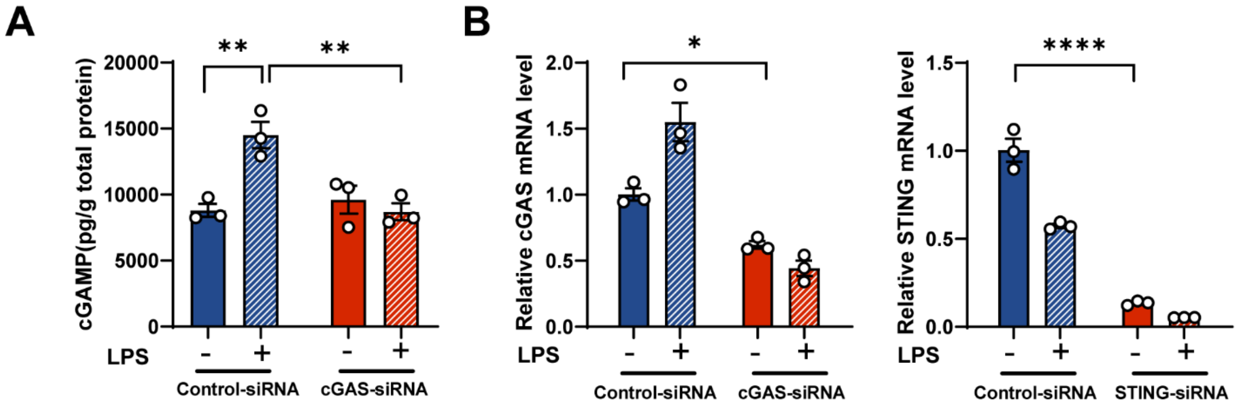

Figure S2. cGAMP synthesis induced by LPS was decreased in cGAS knockout cells.

A-B) HBE cells were transfected with control-siRNA, cGAS-siRNA or STING-siRNA for 24 h and incubated with 100 μg/ml LPS for an additional 24 h. **A**) ELISA analysis to measure cGAMP levels in HBE cells (n=3). **B**) Knockdown efficiency of cGAS(left) and STING (right) siRNA in HBE cells by RT-qPCR (n=3). In (A), (B), the data presented are one representative experiment of three independent experiments and shown as mean ± SEM. Statistical analyses were calculated using two-way ANOVA with Sidaks multiple comparisons. *$p < 0.05$, **$p < 0.01$, ****$p < 0.0001$.

Figure S3

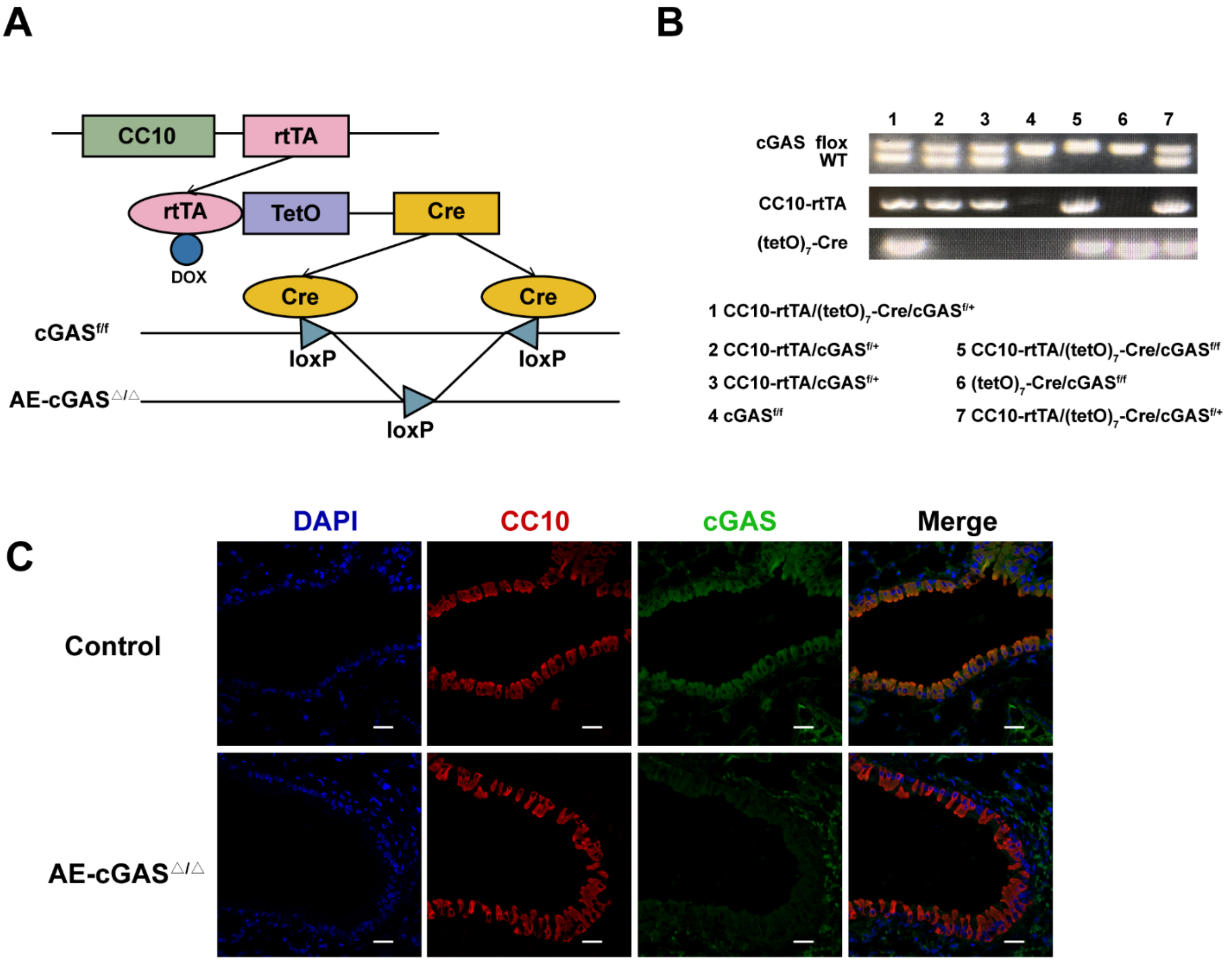

Figure S3. The cell-specific knockdown effects of cGAS in mouse lung tissues.

CC10-rtTA/(tetO)7-Cre/cGAS$^{flox/flox}$ mice and their littermate controls were fed with doxycycline in their drinking water (2 mg/ml) for 20 days (designated as AE-cGAS$^{\triangle/\triangle}$ mice). **A**) Schematic map of the generation of AE-cGAS$^{\triangle/\triangle}$ mice. **B**) Genotyping was analyzed by PCR using genomic DNA from mouse tails. **C**) Representative lung immunofluorescence images of cGAS (green), nuclei (DAPI, blue), and airway epithelial cells (with CC10, red) in AE-cGAS$^{\triangle/\triangle}$ and their littermate controls. Scale bars, 20 μm.

Figure S4

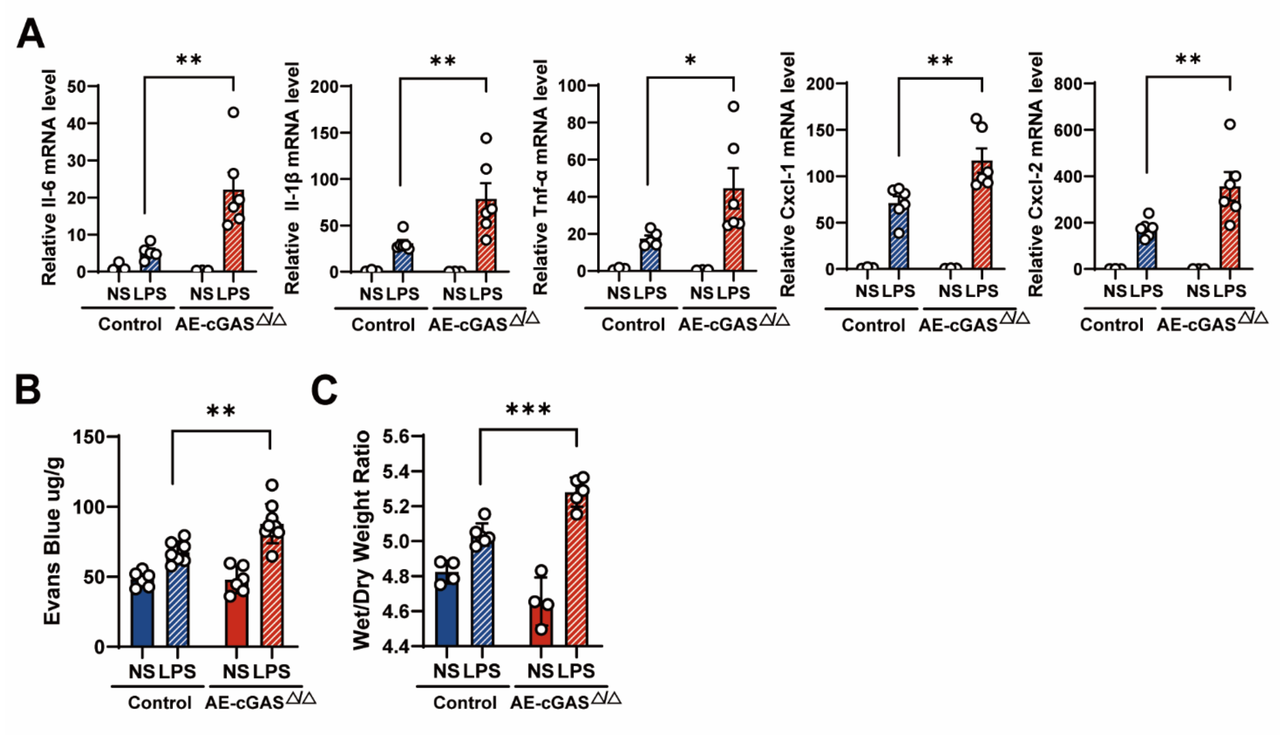

Figure S4. Conditional deletion of cGAS in airway epithelium exacerbates LPS-induced lung injury in vivo.

CC10-rtTA/(tetO)7-Cre/cGAS $^{flox/flox}$ mice and their littermate controls were fed with doxycycline in their drinking water (2 mg/ml) for 20 days (designated as AE-cGAS$^{\Delta/\Delta}$ mice) and then intratracheally challenged with LPS (1 mg/kg) for 24 h. **A**) RT-qPCR of IL-6, IL-1β, TNF-α, CXCL1, and CXCL2 in lung tissues from mice. **B**) Alveolar-capillary permeability of mice. **C**) Wet to dry ratio of the lungs from mice. In (A), (B) and (C), each symbol represents an individual mouse(n=3~8). The data presented are one representative experiment of three independent experiments and shown as mean ± SEM. Two-way ANOVA with Sidaks multiple comparisons. *p < 0.05, **p < 0.01, ***p <0.001.

Figure S5

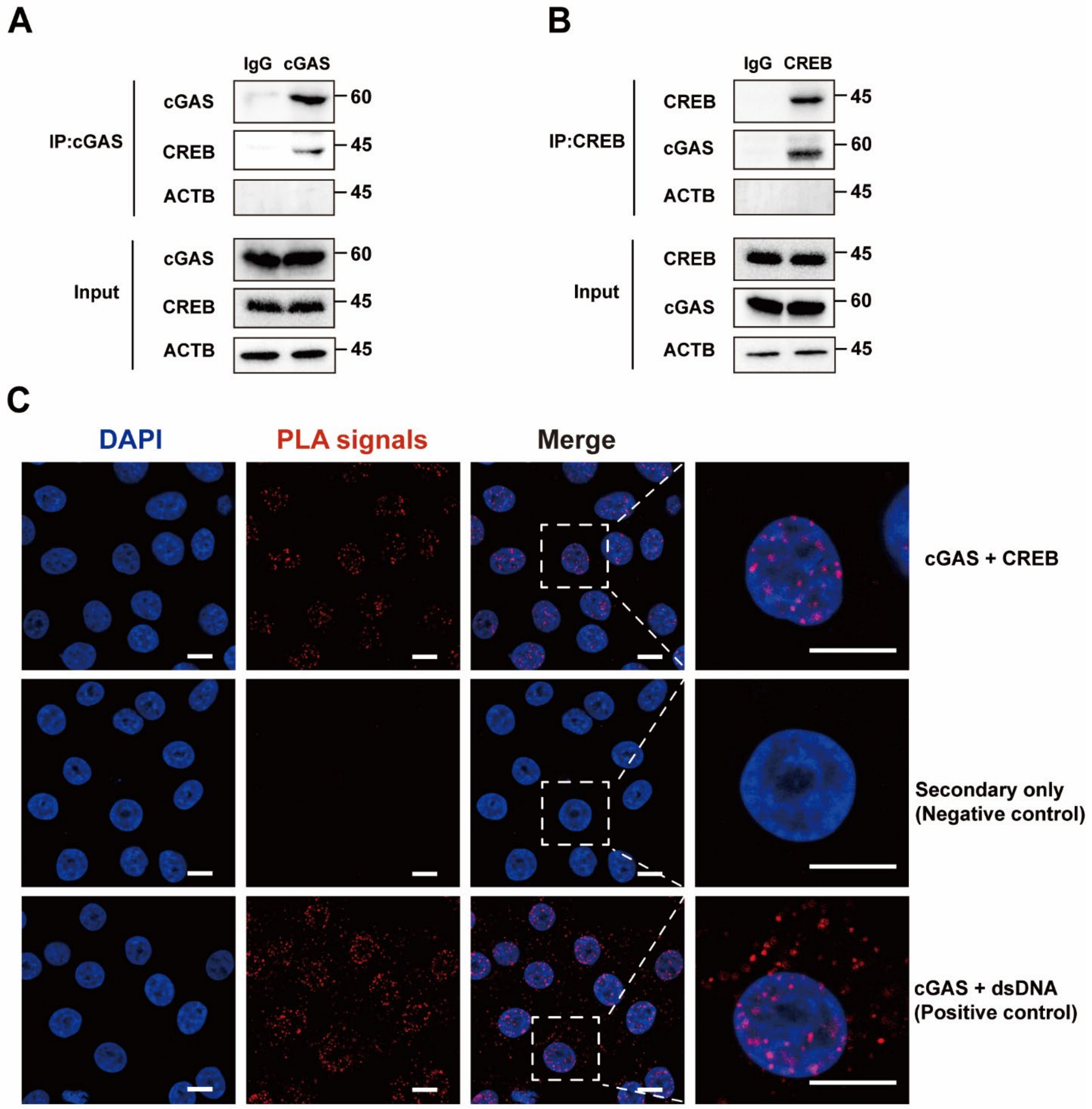

Figure S5. Endogenous cGAS interacts with CREB.

A-B) HBE Cell lysates were immunoprecipitated with anti-cGAS (**A**) or anti-CREB (**B**) and subjected to immunoblotting. **C**) HBE cells were analyzed for the spatial approximation of cGAS with CREB by proximity ligation assay (PLA). Red, proximity ligation-positive signals. Negative control without primary antibodies. Positive control using cGAS with dsDNA by PLA. Scale bars, 10 µm.

Figure S6

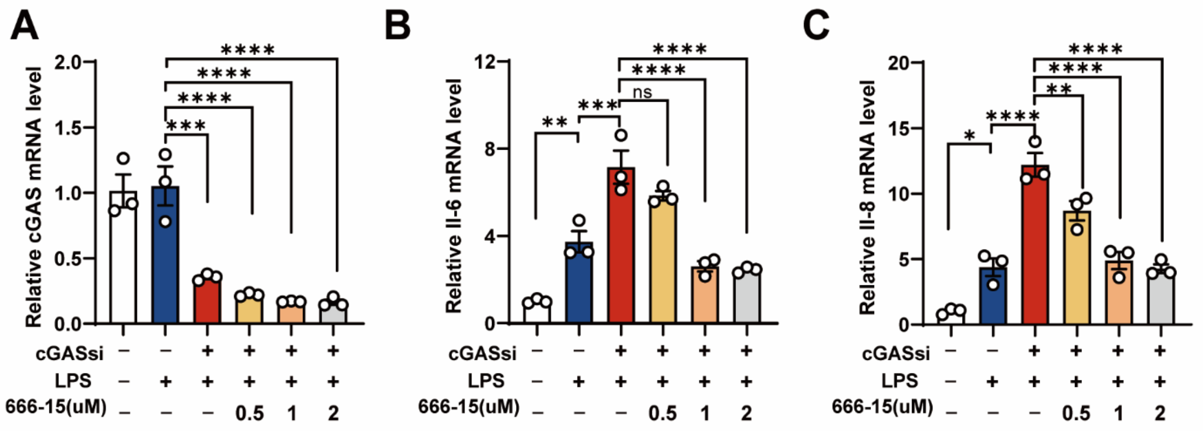

Figure S6. Inhibition of CREB reduces the LPS-induced inflammatory response in cGAS- deficiency airway epithelium in vivo and in vitro.

A-C) HBE cells were transfected with control-siRNA or cGAS-siRNA for 24 h and pretreated with indicated (0, 0.5, 1, 2uM) 666-15(CREB inhibitor) for 2h and subsequent treated with 100 µg/ml LPS for an additional 24 h.**A**) Knockdown efficiency of cGAS siRNA in HBE cells by RT-qPCR (n=3). **B-C**) RT-qPCR of IL-6(B) and IL-8 (C) in HBE cells (n=3). In (A)-(C), the data shown as mean ± SEM and statistical analyses were calculated using one-way ANOVA. The data presented are one representative experiment of three independent experiments. ns, no significance, *p < 0.05, **p < 0.01, ***p < 0.001, ****p <0.0001.
