# Supplementary material for: Airway epithelial cGAS inhibits LPS-induced acute lung injury through CREB signaling
Source: Cell Death Dis. 2023 Dec 19;14(12):844. doi: 10.1038/s41419-023-06364-0 (PMC10730695; doi:10.1038/s41419-023-06364-0)
Supplement: Supplementary file 2 — Original western blots [file 41419_2023_6364_MOESM2_ESM.pdf]

Figure1C

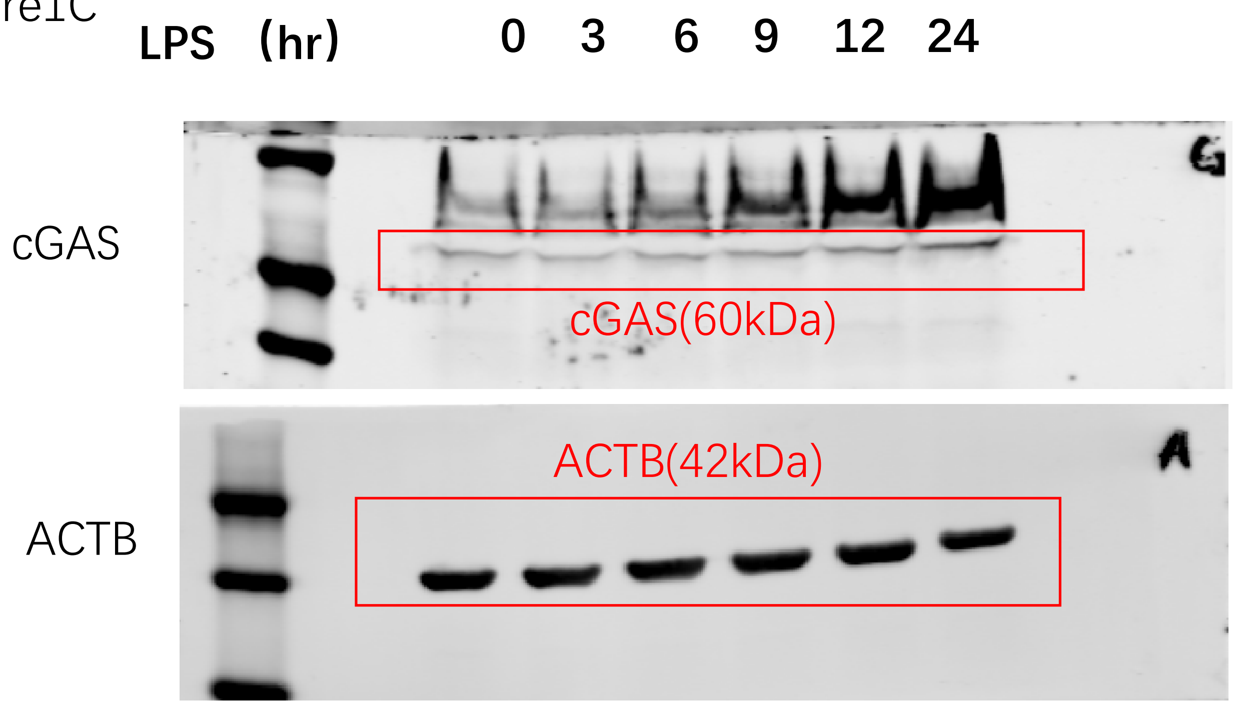

Figure1D

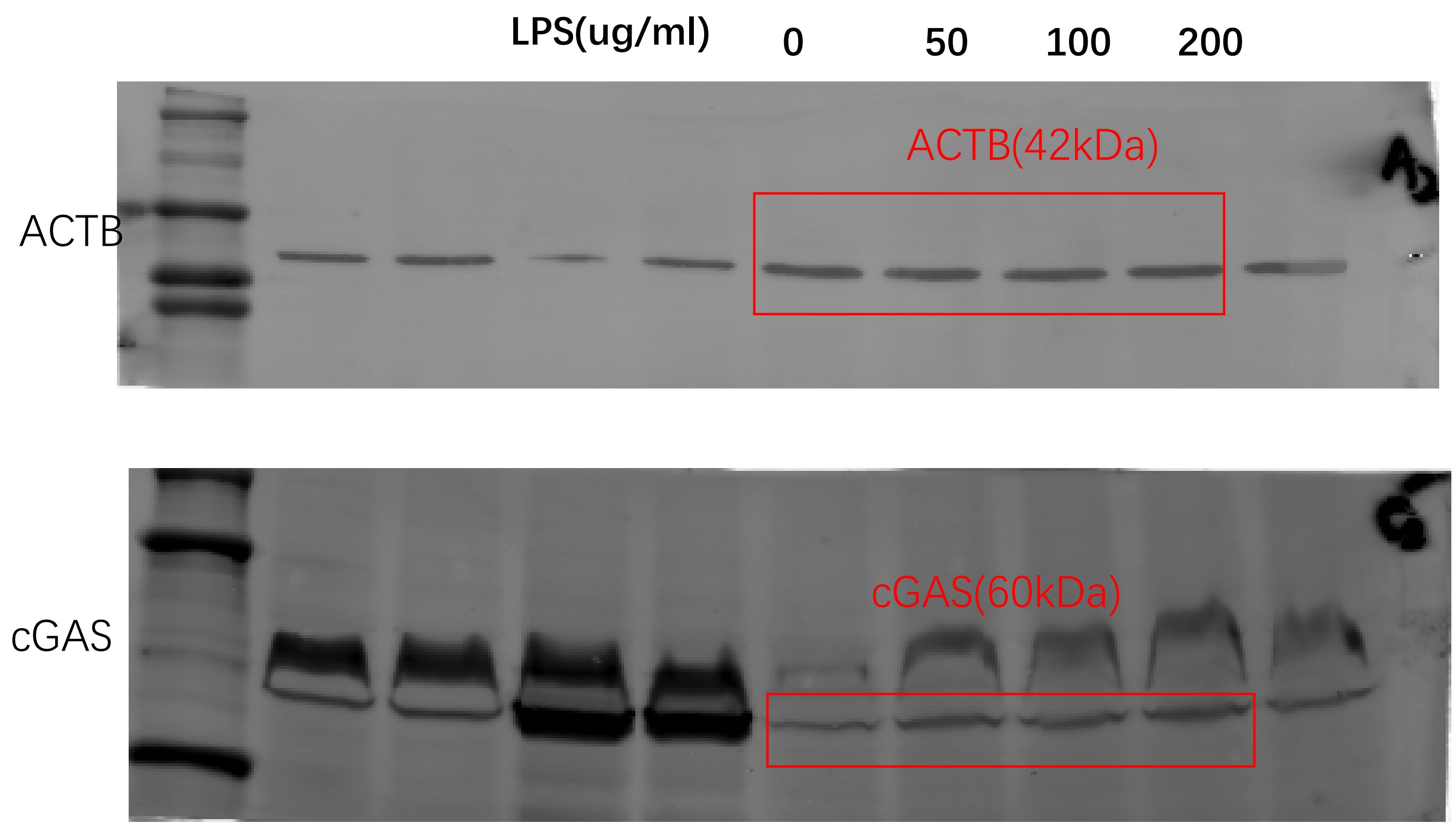

Figure1E

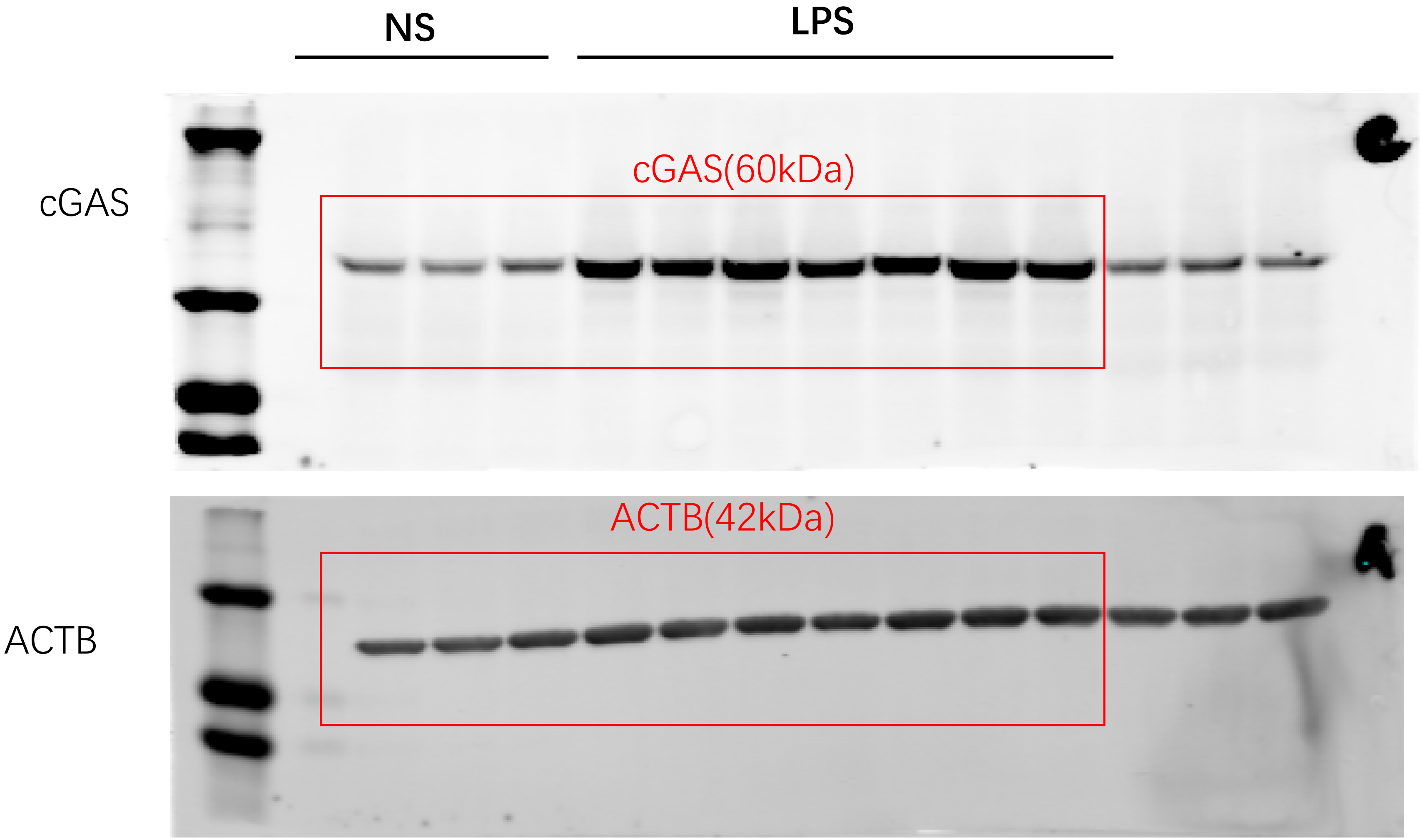

Figure4F

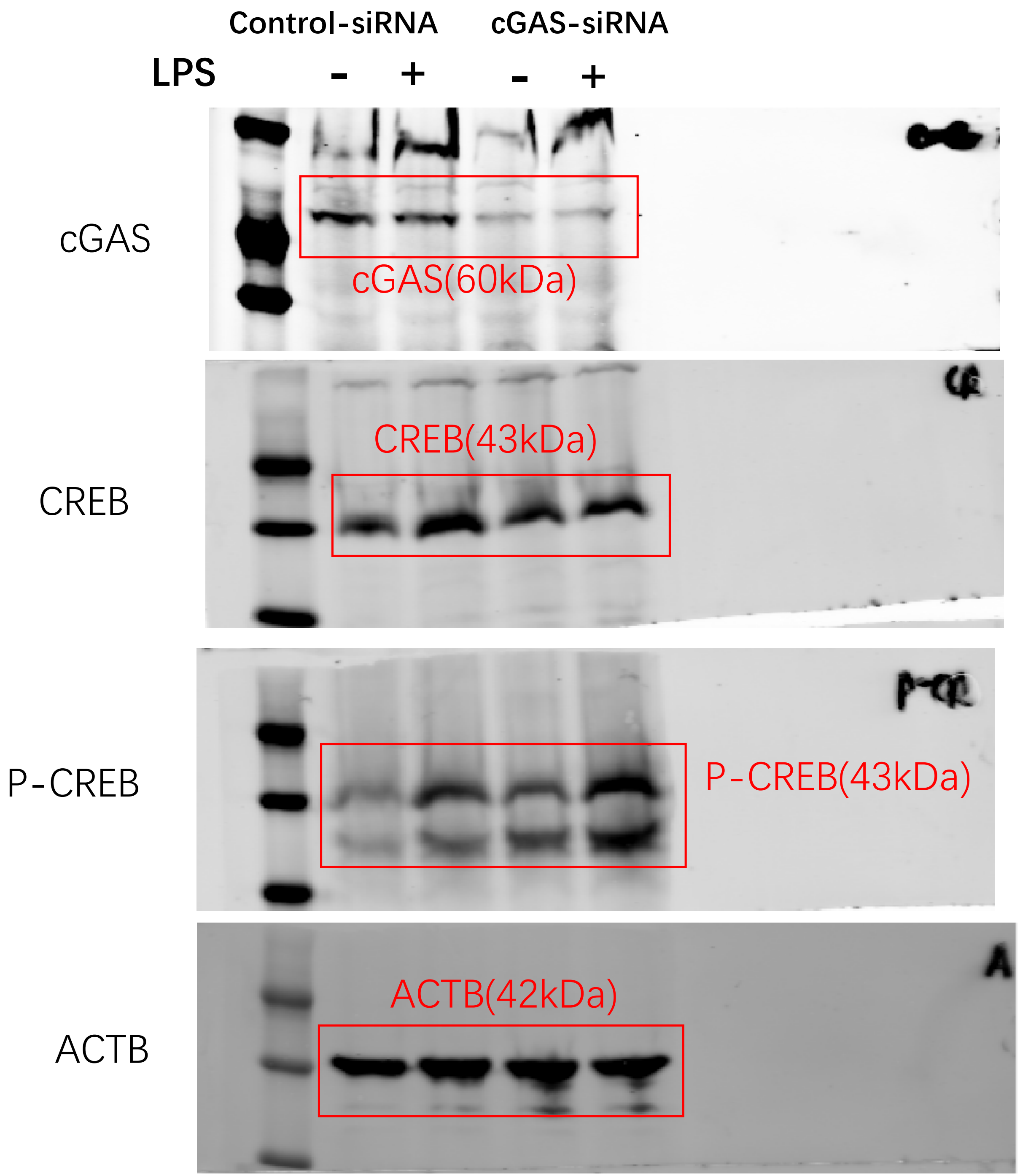

Control-siRNA
cGAS-siRNA
LPS
-
+
-
+
cGAS
cGAS(60kDa)
CREB
CREB(43kDa)
P-CREB
P-CREB(43kDa)
ACTB
ACTB(42kDa)

Figure5A

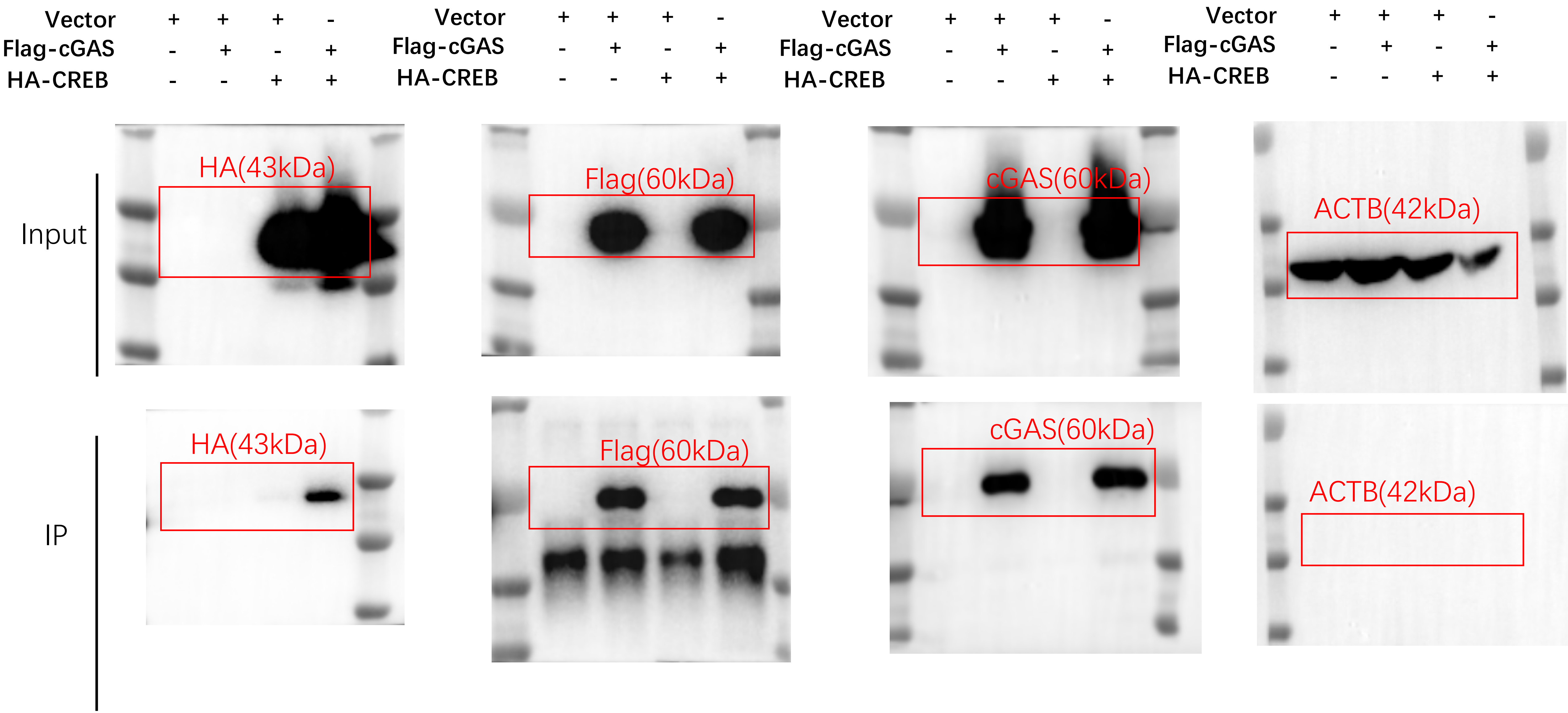

Vector + + + -
Flag-cGAS - + - +
HA-CREB - - + +
Input
IP
HA(43kDa)
Flag(60kDa)
cGAS(60kDa)
ACTB(42kDa)

Figure5B

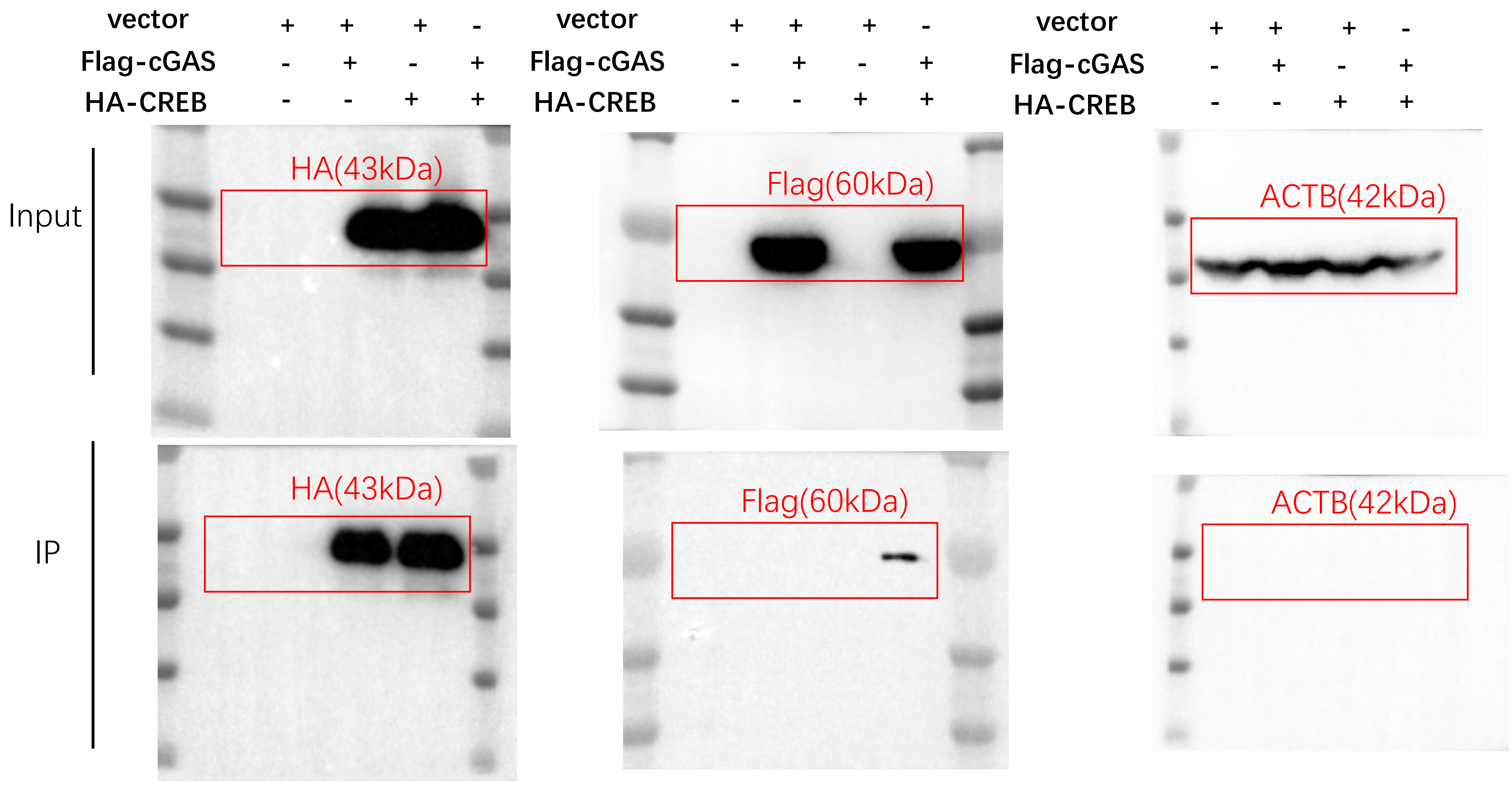
vector + + + -
Flag-cGAS - + - +
HA-CREB - - + +
vector + + + -
Flag-cGAS - + - +
HA-CREB - - + +
vector + + + -
Flag-cGAS - + - +
HA-CREB - - + +
Input
HA(43kDa)
Flag(60kDa)
ACTB(42kDa)
IP
HA(43kDa)
Flag(60kDa)
ACTB(42kDa)

Figure5C

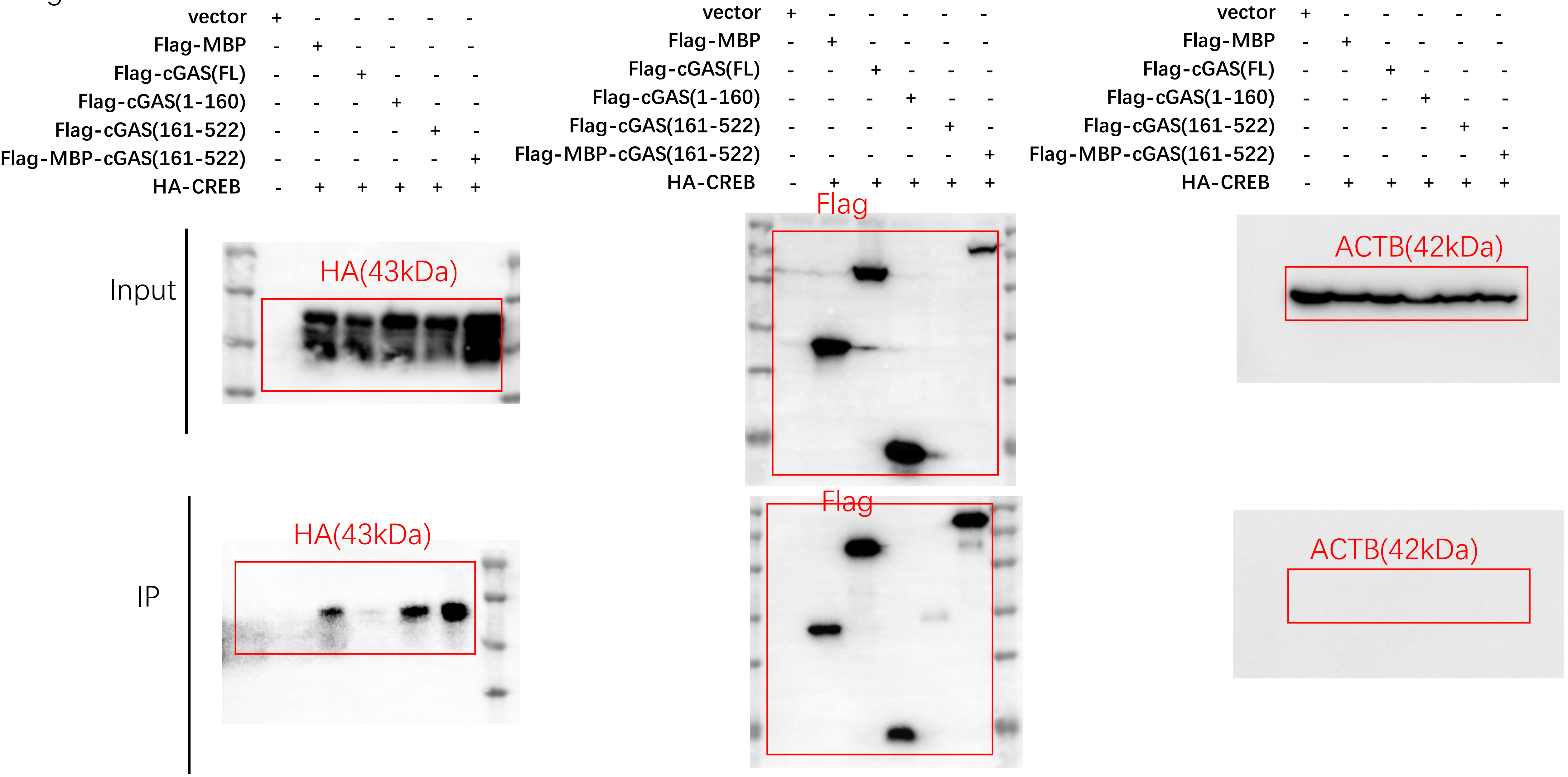
vector + - - - - -
Flag-MBP - + - - - -
Flag-cGAS(FL) - - + - - -
Flag-cGAS(1-160) - - - + - -
Flag-cGAS(161-522) - - - - + -
Flag-MBP-cGAS(161-522) - - - - - +
HA-CREB - + + + + +
Input
IP
HA(43kDa)
HA(43kDa)
Flag
Flag
ACTB(42kDa)
ACTB(42kDa)

FigureS1 B

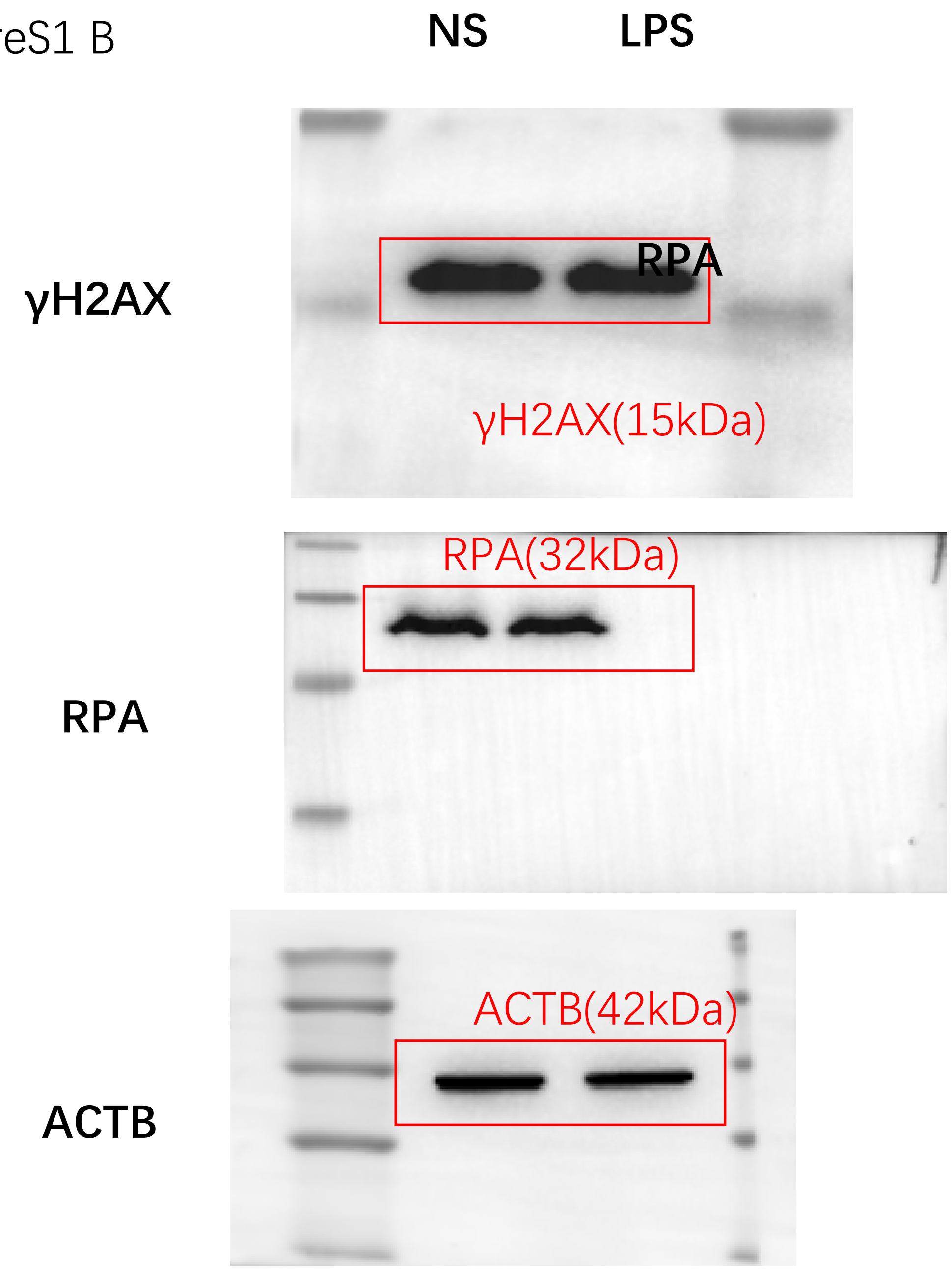

FigureS5A

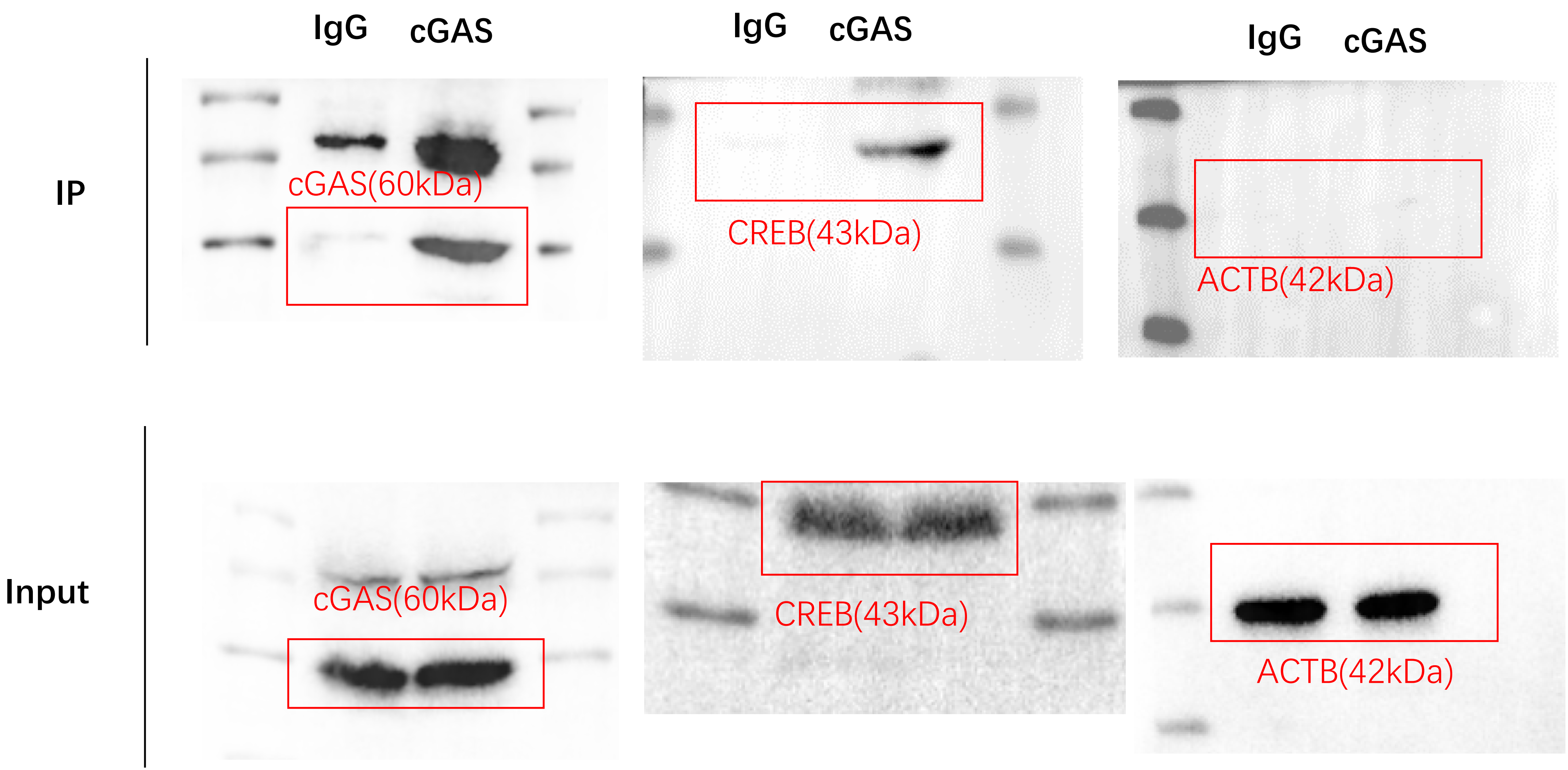
IgG
cGAS
IgG
cGAS
IgG
cGAS
IP
cGAS(60kDa)
CREB(43kDa)
ACTB(42kDa)
Input
cGAS(60kDa)
CREB(43kDa)
ACTB(42kDa)

FigureS5 B

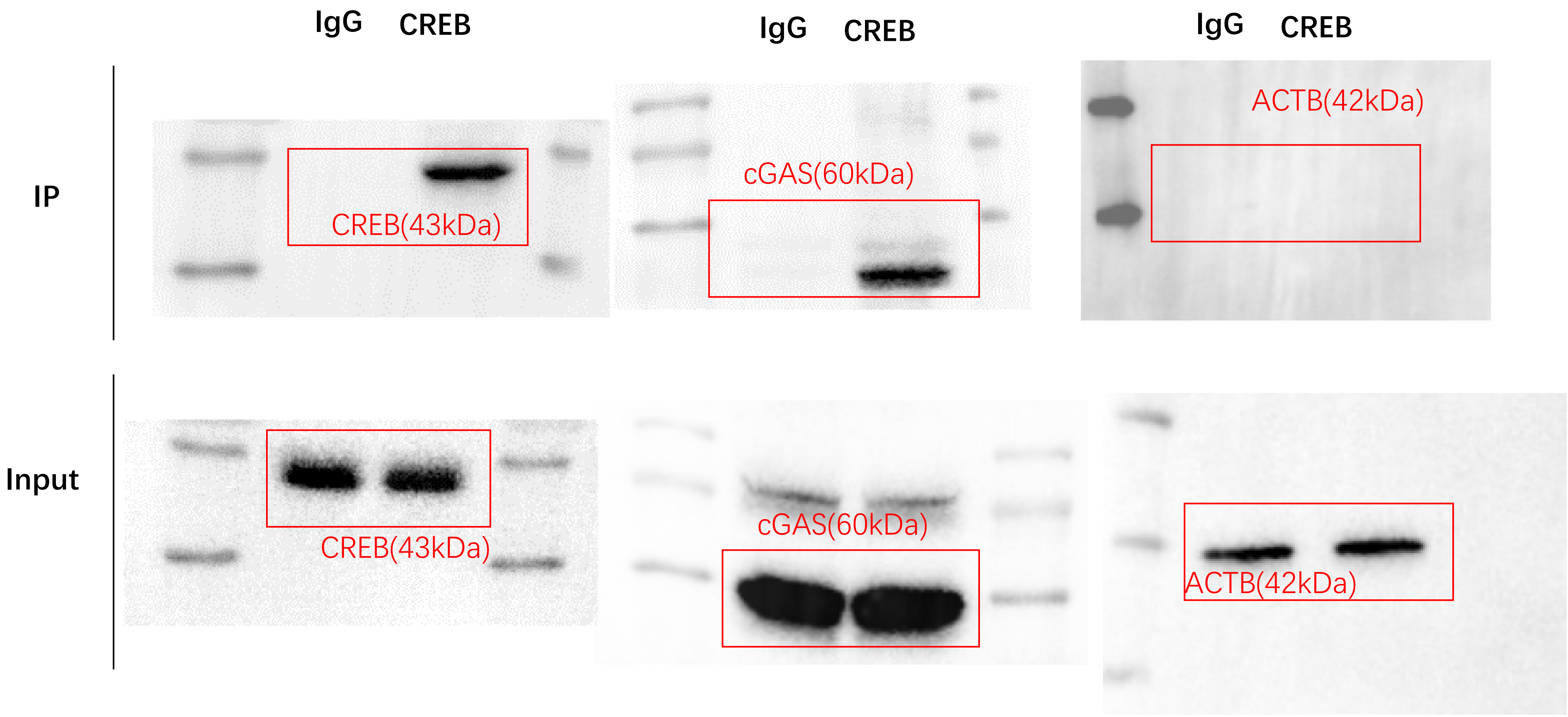
IgG
CREB
IgG
CREB
IgG
CREB
IP
CREB(43kDa)
cGAS(60kDa)
ACTB(42kDa)
Input
CREB(43kDa)
cGAS(60kDa)
ACTB(42kDa)
